# Integrating Behavioral Science and Design Thinking to Develop Mobile Health Interventions: Systematic Scoping Review

**DOI:** 10.2196/35799

**Published:** 2022-03-16

**Authors:** Paula Voorheis, Albert Zhao, Kerry Kuluski, Quynh Pham, Ted Scott, Peter Sztur, Nityan Khanna, Mohamed Ibrahim, Jeremy Petch

**Affiliations:** 1 Institute of Health Policy, Management and Evaluation Dalla Lana School of Public Health University of Toronto Toronto, ON Canada; 2 Centre for Data Science and Digital Health Hamilton Health Sciences Hamilton, ON Canada; 3 Master of eHealth Program McMaster University Hamilton, ON Canada; 4 Institute for Better Health Trillium Health Partners Toronto, ON Canada; 5 Centre for Global eHealth Innovation Techna Institute University Health Network Toronto, ON Canada; 6 Faculty of Health Sciences School of Nursing McMaster University Hamilton, ON Canada; 7 Division of Cardiology Department of Medicine McMaster University Hamilton, ON Canada; 8 Population Health Research Institute Hamilton, ON Canada

**Keywords:** behavior change, design thinking, digital health, health behavior, mobile application, mobile health, mobile phone, product design, scoping review, systems design, telemedicine, user-centered design

## Abstract

**Background:**

Mobile health (mHealth) interventions are increasingly being designed to facilitate health-related behavior change. Integrating insights from behavioral science and design science can help support the development of more effective mHealth interventions. Behavioral Design (BD) and Design Thinking (DT) have emerged as best practice approaches in their respective fields. Until now, little work has been done to examine how BD and DT can be integrated throughout the mHealth design process.

**Objective:**

The aim of this scoping review was to map the evidence on how insights from BD and DT can be integrated to guide the design of mHealth interventions. The following questions were addressed: (1) what are the main characteristics of studies that integrate BD and DT during the mHealth design process? (2) what theories, models, and frameworks do design teams use during the mHealth design process? (3) what methods do design teams use to integrate BD and DT during the mHealth design process? and (4) what are key design challenges, implementation considerations, and future directions for integrating BD and DT during mHealth design?

**Methods:**

This review followed the Joanna Briggs Institute reviewer manual and PRISMA-ScR (Preferred Reporting Items for Systematic Reviews and Meta-Analyses Extension for Scoping Reviews) checklist. Studies were identified from MEDLINE, PsycINFO, Embase, CINAHL, and JMIR by using search terms related to mHealth, BD, and DT. Included studies had to clearly describe their mHealth design process and how behavior change theories, models, frameworks, or techniques were incorporated. Two independent reviewers screened the studies for inclusion and completed the data extraction. A descriptive analysis was conducted.

**Results:**

A total of 75 papers met the inclusion criteria. All studies were published between 2012 and 2021. Studies integrated BD and DT in notable ways, which can be referred to as “Behavioral Design Thinking.” Five steps were followed in Behavioral Design Thinking: (1) empathize with users and their behavior change needs, (2) define user and behavior change requirements, (3) ideate user-centered features and behavior change content, (4) prototype a user-centered solution that supports behavior change, and (5) test the solution against users’ needs and for its behavior change potential. The key challenges experienced during mHealth design included meaningfully engaging patient and public partners in the design process, translating evidence-based behavior change techniques into actual mHealth features, and planning for how to integrate the mHealth intervention into existing clinical systems.

**Conclusions:**

Best practices from BD and DT can be integrated throughout the mHealth design process to ensure that mHealth interventions are purposefully developed to effectively engage users. Although this scoping review clarified how insights from BD and DT can be integrated during mHealth design, future research is needed to identify the most effective design approaches.

## Introduction

### Background

Digital health interventions are increasingly being designed to help people manage their health [1]. Many of these digital health interventions seek to facilitate behavior change and are often referred to as “digital behavior change interventions” (DBCIs) [2]. Among the wide range of DBCIs available, mobile health (mHealth) interventions have the potential to improve the reach and efficiency of health support owing to the widespread use of mobile phones [3,4]. Despite the potential impact of mHealth DBCIs, there is mixed evidence on whether they are effective at changing health behavior and improving health outcomes [3]. One concern is that patients and the public struggle to “effectively engage” with mHealth DBCIs [5,6]. Effective engagement with mHealth DBCIs has been defined as necessitating both microengagement with the mHealth interface itself (eg, logging into the app, entering data) and macroengagement with the behavior changes the mHealth intervention aims to support (eg, performing the exercises prescribed by the app) [6]. Therefore, for an mHealth DBCI to “effectively engage” users, it must be designed with engaging user-centered features to support microengagement and evidence-based behavior change techniques (BCTs) to support macroengagement.

To design effective behavior change content, there is evidence that mHealth interventions developed using the behavior change theory and BCTs are more likely to be effective than those without [7]. Behavioral scientists have been developing methods to systematically transition from diagnosing a behavioral problem to designing a behavior change intervention [8-11], which can be referred to as Behavioral Design (BD) [12]. Bondaronek and colleagues [13] provide several examples of how BD can be operationalized in publicly available physical activity apps. For instance, the app “Movesum” uses BCT 1.1 Goal Setting (Behavior) in the form of an easily adjustable step count goal [13,14]. BCT 1.1 Goal Setting (Behavior) is particularly useful when users struggle to plan for what they want to achieve or how they want to act [15]. Overall, BD can be operationalized in diverse ways but generally involves the following steps: (1) understanding the behavioral problem, (2) making a behavioral diagnosis for the target behavior using behavioral theories, models, and frameworks, (3) identifying relevant BCTs using taxonomies and classifications, (4) translating BCTs into intervention features, and (5) evaluating behavior change outcomes.

To design engaging user-centered mHealth features, there is evidence that mHealth interventions developed using person- and user-centered design processes are more likely to facilitate user engagement and improve intervention effectiveness [16,17]. Person- and user-centered design processes vary in terms of their operationalization but always put user needs at the forefront of design. Design Thinking (DT) is a common framework used in design science to guide creative user-centric designs for mHealth [18]. Design thinkers can use a range of approaches during the design process to ensure user-centeredness, such as directly involving users in app development or referring to Nielsen’s 10 usability heuristics [19]. The Nielsen Norman Group provides several detailed examples of how different usability principles can be implemented in application design [20,21]. Overall, DT can be executed in different ways but generally involves the following steps: (1) empathizing with the user, (2) defining the user requirements, (3) ideating functional concepts, (4) prototyping the user-centered solution, and (5) testing the solution to see if users’ needs are met. Despite knowledge about what constitutes “effective engagement” with mHealth DBCIs, a recent scoping review of DBCIs developed over the past 2 decades found that most design teams make limited use of BCTs and do not adequately describe the methods they employ to meet users’ needs [22]. This scoping review concluded that DBCI practitioners have little guidance on how to integrate best practices from behavioral science and design science, and a need exists to develop guidance to support them through this process [22]. This conclusion has been reiterated across the literature, with experts agreeing that methodological guidance is required to design effectively engaging DBCIs [23].

Experts have also begun to discuss similarities and differences between approaches used by design scientists and behavioral scientists [24-26]. For example, design scientists often rely on end users to ideate content based on their stated preferences, needs, and recommendations. They iteratively build interventions by using ongoing feedback, with a focus on producing creative solutions that users will enjoy (ie, ensuring the users are microengaged). In contrast, behavioral scientists focus on producing solutions that will nudge behavior change (ie, ensuring the users are macroengaged). Behavioral scientists often rely on theory- and evidence-based linkages to understand behavioral problems and select intervention content. They rigorously test solutions against their ability to effect behavior change and not necessarily how and whether the user is engaging with them. Nonetheless, both approaches emphasize the importance of understanding and diagnosing the problem at hand before proposing, designing, and implementing a solution. Both approaches also aim to ensure that the resulting solutions are designed purposefully to achieve user engagement. Amalgamating best practices from DT and BD may be mutually beneficial and help design teams develop more “effectively engaging” DBCIs [24-26]. There is currently a knowledge gap with respect to how best practices from DT and BD can be integrated to develop “effectively engaging” mHealth DBCIs. Specifically, little is known about how DT and BD can be blended throughout the mHealth DBCI design process to ensure that microengagement and macroengagement needs will be met.

### Aims and Objectives

The aim of this scoping review was to identify and map how design teams have integrated best practices from BD and DT throughout the mHealth DBCI design process. By clarifying how BD and DT can be integrated, this review aimed to provide guidance on how mHealth DBCIs can be designed to more “effectively engage” patients and the public. This scoping review addressed the following questions: (1) what are the main characteristics of the studies that integrated BD and DT during the mHealth DBCI design process? (2) what theories, models, and frameworks did design teams use during the mHealth DBCI design process? (3) what methods did design teams use to integrate BD and DT during the mHealth DBCI design process? and (4) what are the key design challenges, implementation considerations, and future directions for the integration of BD and DT during mHealth DBCI design?

## Methods

### Study Design

The Joanna Briggs Institute reviewer’s manual was used to guide the conduct of this scoping review [27]. The scoping review follows the PRISMA-ScR (Preferred Reporting Items for Systematic Reviews and Meta-Analyses Extension for Scoping Reviews) checklist (see Multimedia Appendix 1) [28]. A scoping review protocol was drafted internally among key stakeholders, including mHealth software architects, mHealth design team managers, an information specialist, and several researchers with experience in the topic.

### Search Strategy

The search strategy was developed by the lead author in collaboration with an information specialist. Initial searches were conducted in journals highly relevant to our research topic (eg, Cochrane, JMIR journals) to identify suitable search terms. A series of initial searches in MEDLINE were completed to analyze the text words contained in the title and abstracts of retrieved papers and index terms used to describe the papers. A final list of search terms was compiled, and a search of the databases MEDLINE, PsycINFO, EMBASE, and CINAHL was completed on May 15, 2021. A handsearch of *Journal of Medical Internet Research* was also completed on May 15, 2021, as this was recognized as a journal highly relevant to the research topic. A gray literature search and a review of paper reference lists were not conducted. This decision was made to tighten the scope of the review around the research objectives, given time and resource constraints. Search terms combined the following topics: mHealth, behavior change, and design thinking. The full search strategy can be seen in Multimedia Appendix 2.

### Eligibility Criteria

Included papers had to be primary studies, where a full-text paper described the design process of an mHealth DBCI. More specifically, included papers had to describe how BD and DT practices were integrated throughout the design of an mHealth DBCI that aimed to support behavior change in patients or the public. To meet these criteria, the papers had to clearly describe their mHealth DBCI design process, addressing at least 3 of the 5 design process steps suggested in DT (empathizing, defining, ideating, prototyping, and testing) [18]. In addition, papers had to clearly describe how behavior change theories, models, frameworks, or techniques were incorporated into the mHealth design process. Studies that only used behavior change insights to evaluate the mHealth DBCI after development were excluded. Furthermore, studies that described an mHealth intervention designed to provide a psychological treatment (eg, cognitive behavioral therapy) without describing how their design process utilized behavior change theories, models, and frameworks to support “effective engagement” were also excluded. No limitations were put on the year of publication; however, only papers published in English were included. To be eligible, the intervention must have been an mHealth intervention, defined by the World Health Organization as “health care and public health practice supported by mobile devices such as mobile phones, tablets, patient monitoring devices, and other wireless devices” [4]. The intervention must also have been designed for use by patients or the public. If the intervention was designed only for use by health care professionals, it was excluded.

### Evidence Selection

Studies from the database searches were handled using Covidence (Veritas Health Innovation Ltd) reference management software. Papers were deduplicated and imported for screening using Covidence. A 2-level screening was performed after duplicate removal. During level 1 screening, titles and abstracts were screened using the eligibility criteria. Publications with title or abstract not meeting the eligibility criteria were excluded. During level 2 screening, full-text papers that passed level 1 were screened. Studies that met the eligibility criteria were included for full data extraction. Consistent with PRISMA-ScR, reasons for exclusion were recorded at the full-text level [28]. Prior to the selection of sources, 2 reviewers completed a pilot screening of 50 titles and abstracts to assess the reliability of the eligibility criteria. Interrater agreement for study inclusion was calculated using percentage agreement. If agreement was lower than 80%, the eligibility criteria would be clarified and another pilot test would occur. All interrater discrepancies during level 1 and 2 evidence selection were resolved between the 2 reviewers upon discussion. The 2 reviewers screened all titles, abstracts, and full-text papers for inclusion.

### Data Extraction

The reviewers extracted data from the eligible papers by using 2 data extraction forms. The first data extraction form elicited the main study characteristics, including lead author, year of publication, journal of publication, country of origin, study design, study purpose, target user population, the health issue, the target health behavior, mHealth DBCI summary, mHealth DBCI design process duration, and the members of the mHealth DBCI design team. The second data extraction form elicited details about how BD and DT were integrated throughout the design process, which included extracting types of theories, models, and frameworks used; the approaches design teams used to integrate best practices from BD with DT over the course of the mHealth DBCI design process, key challenges in the mHealth DBCI design process, key implementation considerations for mHealth DBCIs, and future considerations for the mHealth DBCI design. The data extraction forms were drafted, revised, and agreed upon by the 2 reviewers after an iterative process of implementing the extraction forms on a sample of papers.

### Analysis and Presentation of Results

A descriptive analysis of the included papers was conducted to meet the objectives of the scoping review. Narrative descriptions, frequency calculations, and visual diagrams were utilized to communicate the results.

## Results

### Evidence Selection

**Figure 1:**
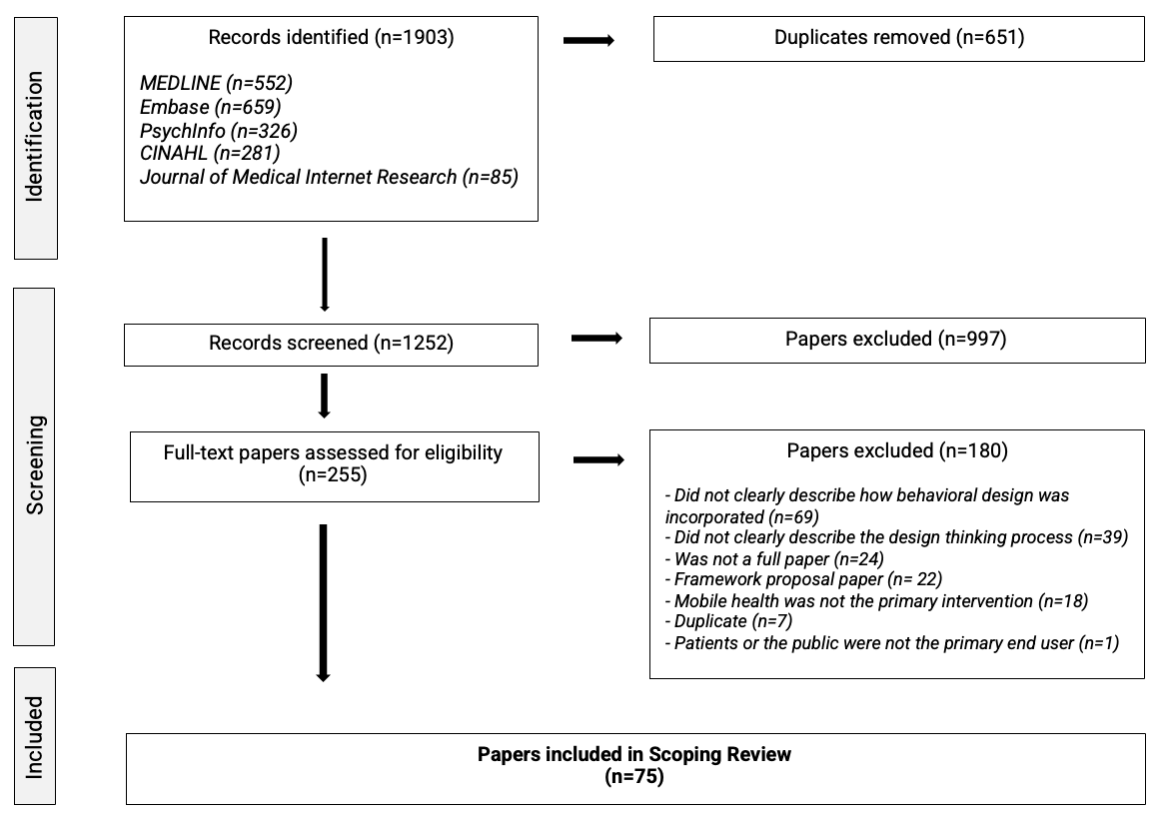
PRISMA-ScR (Preferred Reporting Items for Systematic Reviews and Meta-Analyses Extension for Scoping Reviews) flow diagram of the study selection process.

A total of 1912 papers were identified from the searches. After 651 duplicate studies were removed, 1252 papers were screened based on their titles and abstracts, with 255 full-text papers meeting the eligibility criteria. After full-text review, 75 papers fulfilled the eligibility criteria and were included in the final review. Figure 1 shows the PRISMA-ScR flow diagram illustrating the paper selection process [28].

### Main Characteristics of the Included Papers

All 75 primary studies were published between 2012 and 2021, with a surge from 2018 onward (51/75, 68%). The Journal of Medical Internet Research and its sister journals accounted for almost 55% (41/75) of the papers included. The United States (14/75, 19%), the United Kingdom (13/75, 17%), Australia (10/75, 13%), and Holland (9/75, 12%) were the most common study locations. The target population of the mHealth DBCIs varied, with the most common being patients with cardiovascular issues (7/75, 9%), patients with diabetes (5/75, 7%), adults with overweight and obesity (5/75, 7%), adults who smoke (5/75, 7%), adults with poor physical activity levels (5/75, 7%), and cancer survivors (5/75, 7%). The target health behaviors addressed also varied, with the most prominent being improved physical activity (18/75, 24%), improved diet (17/75, 23%), disease self-management (12/75, 16%), preventative health behaviors (6/75, 8%), adherence to medication (5/75, 7%), adherence to rehabilitation programming (5/75, 7%), and smoking cessation (5/75, 7%). mHealth DBCI design teams were multidisciplinary in their membership. Members included researchers, patients, caregivers, community partners, clinicians, technology developers, and experts in behavior change, health psychology, health communications, health promotion, and health informatics. Topic experts were also relied on depending on the type of intervention (eg, diabetes educators). Software engineers, computer scientists, videographers, product designers, and graphic designers were brought to assist with the development of the mHealth intervention, although it was usually unclear when they were included. The design process duration was usually not reported. Out of the 14 interventions that clearly reported design process duration, there was a large variation in timespan, ranging from less than 3 months to upwards of 4 years. Multimedia Appendix 3 provides an overview of the main study characteristics, and Multimedia Appendix 4 provides a full list of the 75 studies included.

### Theories, Models, and Frameworks Used During Design

**Figure 2:**
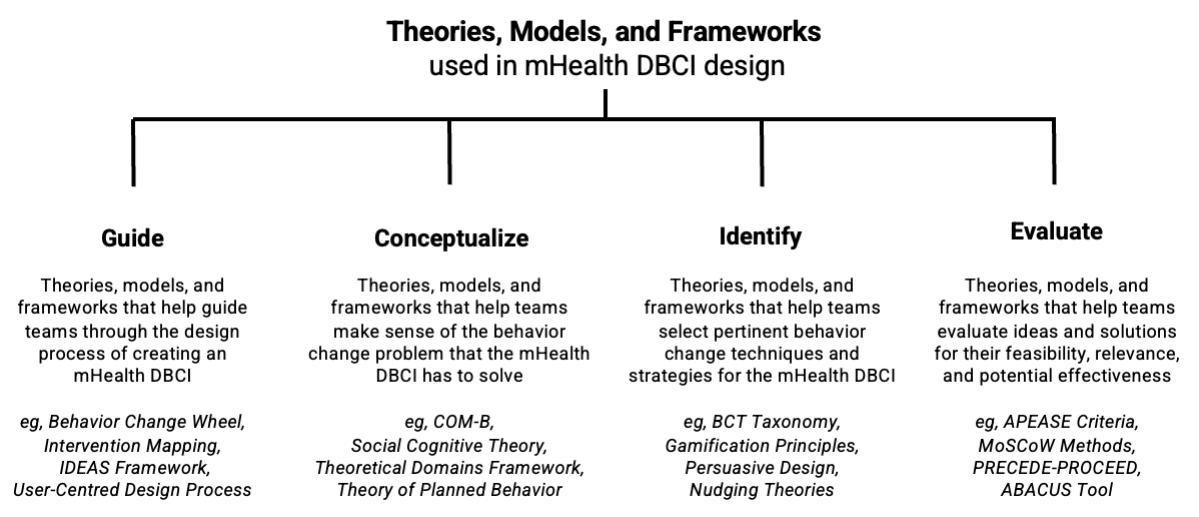
Theories, models, and frameworks used in mobile health digital behavior change intervention design. ABACUS: App Behavior Change Scale; APEASE: Acceptability, Practicability, Effectiveness, Affordability, Side-effects, and Equity; BCT: behavior change technique; COM-B: capability, opportunity, motivation-behavior; DBCI: digital behavior change intervention; IDEAS: Integrate, Design, Assess, and Share; mHealth: mobile health; MoSCoW: must-have, should-have, could-have, and won't-have, or will not have right now; PRECEDE: Predisposing, Reinforcing, and Enabling Constructs in Educational Diagnosis and Evaluation; PROCEED: Policy, Regulatory, and Organizational Constructs in Educational and Environmental Development.

Studies used a variety of theories, models, and frameworks in their mHealth DBCI design process. Theories, models, and frameworks were most often used to (1) guide the design process itself, (2) conceptualize the behavior change problem, (3) identify relevant BCTs, and (4) evaluate ideas for their applicability, feasibility, or potential effectiveness. Figure 2 summarizes the types of theories, models, and frameworks used in the mHealth DBCI design process, and Multimedia Appendix 5 provides a detailed breakdown.

### Methods Used to Integrate BD and DT During the mHealth DBCI Design Process

**Figure 3:**
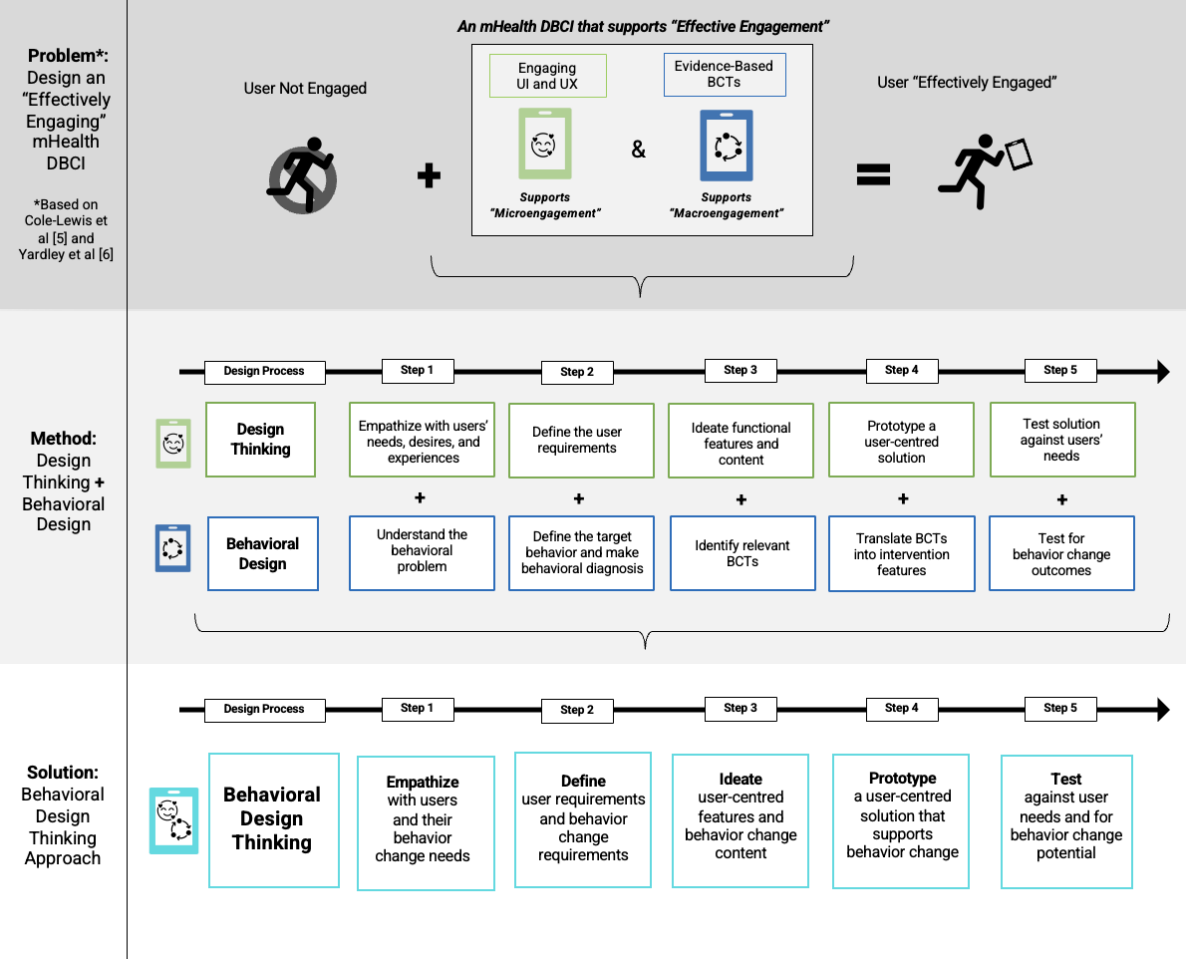
The behavioral design thinking approach [5,6]. BCT: behavior change technique; DBCI: digital behavior change intervention; mHealth: mobile health; UI: user interface; UX: user experience.

Regardless of the theories, models, and frameworks teams used to design their mHealth DBCIs, they integrated best practices from BD and DT in notable ways. We refer to the mixing of BD and DT throughout the mHealth DBCI design process as the “Behavioral Design Thinking Approach” (see Figure 3). The Behavioral Design Thinking Approach presents a new method of designing mHealth DBCIs, which is the result of merging together best practices from BD and DT. Multimedia Appendix 6 provides further detail on the Behavioral Design Thinking Approach along with several specific examples on how BD and DT can be integrated.

Generally, 5 steps are followed in the Behavioral Design Thinking Approach: (1) empathize with users and their behavior change needs, (2) define user and behavior change requirements, (3) ideate user-centered features and behavior change content, (4) prototype a user-centered solution that supports behavior change, and (5) test the solution against users’ needs and for its behavior change potential. Across these steps, studies integrated DT and BD in different ways, often “driving” their mixing in a certain direction. The 3 ways DT and BD were mixed included (1) DT and BD approaches were equally weighted (*notation = DT+BD*), (2) DT drove the approach, with concepts from BD supplementing the DT approaches (*notation = DT→BD*), or (3) BD drove the approach, with concepts from DT supplementing the BD approaches (*notation = BD→DT*). Overall, studies tended to “empathize” by blending DT and BD equally (*DT+BD*), “define” by blending DT and BD equally (*DT+BD*), “ideate” by driving the process by BD (*BD→DT), “*prototype” by driving the process by DT *(DT→ BD*), and “test” by driving the process by DT *(DT→BD).* These approaches are summarized in the text below.

### Behavioral Design Thinking Approach

#### Behavioral Design Thinking Step 1: Empathize With Users and Their Behavior Change Needs

Most studies described how they empathized with users while simultaneously conceptualizing the behavior change problem (66/75, 88%), blending concepts from DT and BD harmoniously *(notation = DT+BD).* To empathize with the users who would perform a target behavior, studies analyzed users’ experiences, perceptions, beliefs, needs, and preferences with their health issues, health behaviors, health interventions, and mobile app usage. To understand the behaviors that the users will perform, studies examined applicable target health behaviors, behavioral determinants, BCTs, and behavioral theories, models, and frameworks. Finally, to understand the context that the users perform the behavior in, studies assessed current practices and programs, relevant personal, social, environmental, and structural factors, and pertinent clinical, usage, and behavioral aims. Studies used primary research (eg, interviews, focus groups, surveys, creative workshops) and secondary research (eg, secondary data analyses, literature reviews, reviews of other interventions, guidelines, practices) to empathize. Studies not only involved patient and public end users during this step (48/75, 64%), but also involved health care practitioners, community partners, behavioral experts, design scientists, and technology developers. To directly involve patients and the public, studies used a variety of tools such as interview guides informed by behavior change models and visual presentations of apps to stimulate collaborative discussion.

#### Behavioral Design Thinking Step 2: Define User and Behavior Change Requirements

Studies tended to analyze the empathy results in order to define system requirements that would meet both users’ needs and behavior change needs *(notation = DT+BD).* Studies tended to define user-centered requirements by sorting users’ stated preferences into key themes to be addressed. Studies tended to define the behavior change requirements by using the empathy results to formulate a “behavioral diagnosis,” which outlines the behavioral determinants that need to be addressed. When studies organized the requirements into an amalgamated format, DT or BD usually drove the organization. For example, some studies used tables organized by the relevant behavioral determinants, whereby they would then list corresponding user quotes and resultant requirements alongside the behavioral determinants. Other studies opted to use the requirements to create holistic user personas and scenarios. Regardless of the approach to amalgamate and make sense of the requirements, the requirement lists were often lengthy and needed to be refined. Refining the requirements involved (1) identifying the requirements that met the project scope and objectives, (2) using feasibility criteria to refine and select certain requirements, (3) consulting experts and patient and public users to prioritize the requirements, and (4) ranking the most important requirements according to their likelihood to elicit behavior change, alignment with current practices, adaptability to a digital interface, acceptability to users, and compatibility with data collection needs.

#### Behavioral Design Thinking Step 3: Ideate User-Centered Features and Behavior Change Content

Most studies described how they translated requirements into design content and features by using ideation methods (67/75, 89%). Generally, studies drove their ideation methods using BD *(notation = BD→DT).* For instance, several studies relied only on BD to ideate the relevant behavior change content (19/67, 28%). These studies used researcher-led “behavioral mapping” to match relevant behavior change determinants with BCTs by using evidence-based taxonomies and tested linkages. It was often unclear, however, exactly how these studies translated the identified BCTs into content for the mobile app. Other studies attempted to completement BD approaches with DT methods during their ideation (48/67, 72%). In these cases, studies would creatively ideate design content and features that would support behavior change while also meeting users’ needs and preferences. These studies relied on different techniques such as design team brainstorming, expert stakeholder panels, technology partner consultation, and end user co-design efforts. Design teams tended to brainstorm how the behavior change insights could be integrated with users’ preferences, existing programming, norms in mHealth design, and clinical management needs. Stakeholder panels would discuss all the requirements and consider the most appropriate strategies (dosage, delivery, organization, and personalization). Technology partners would specify how requirements could be operationalized within an mHealth app. Patient and public partners would contribute to co-design sessions to create and reflect on ideas, content, and designs. Approximately 34% (23/75) of the studies appeared to directly involve patient and public users during this step to ensure that the ideated content met their unique needs.

#### Behavioral Design Thinking Step 4: Prototype a Solution That Is User-Centered and Supports Behavior Change

Many studies did not clearly describe the prototyping methods they used to translate ideated content and features into functional solutions (28/75, 36%). Studies that described their prototyping approach tended to drive their prototyping by using DT (*notation = DT→BD*). For instance, a large proportion of studies only used prototyping methods, tools, and aids traditional to DT (37/47, 79%). DT prototyping methods usually involved iterative prototyping, feedback, and refinement, often utilizing sprint, scrum, or agile methods. DT prototyping tools included wireframing, paper prototyping, application flowcharts, and use case scenarios. DT prototyping aids included tools such as Nielsen’s usability heuristics [19] and the Eight Golden Rules [29]. Despite reliance on DT for prototyping, several studies supplemented DT methods with BD considerations to ensure that behavior change content would be operationalized within the prototyped solution (10/47, 21%). For example, to supplement DT prototyping methods, some studies utilized BCT codevelopment to ensure that BCTs were not lost in translation during technological development. To supplement DT prototyping tools, some studies elected to make BCT flowcharts to clarify how the BCTs would be interacted with. To supplement DT prototyping aids, some studies also used the Behavioral Intervention Technology Model [30] or the Persuasive Systems Design Framework [31], which were behaviorally informed models used to guide feature selection. Among the studies that clearly described their prototyping methods, just over half appeared to directly involve patient and public end users in their prototyping process (26/47, 55%).

#### Behavioral Design Thinking Step 5: Test Solution Against User Needs and for Its Behavior Change Potential

A large proportion of studies did not clearly describe how they tested their mHealth DBCI solution within the design process (18/75, 24%). Studies that described their testing methods tended to drive their testing by methods traditional to DT (*notation =* *DT→BD)*. For instance, a considerable number of studies relied exclusively on DT evaluation approaches such as heuristic evaluation, usability testing, expert evaluation, and pilot testing to evaluate the solutions against users’ needs (33/57, 58%). Despite reliance on DT for testing, some studies supplemented these testing methods with BD considerations to evaluate the solution for its behavior change potential (24/57, 42%). For instance, in addition to traditional DT heuristic evaluation, some studies conducted a BCT evaluation to assess the final solution for the presence of known BCTs. In addition to traditional DT usability testing, some studies “tagged” BCT components within the app to follow how users engaged, accepted, and perceived the intended BCTs. Some studies also conducted posttest user interviews directed by interview guides based on constructs of a behavioral model. In addition to traditional DT expert evaluations, some studies brought on behavioral science experts to assess the extent to which the intervention content had fidelity to the intended BCTs. Experts also could assess the quality of the mHealth DBCI by using the App Behavior Change Scale [32]. If pilot tests were conducted, specific behavior change outcomes could be evaluated in addition to traditional usage metrics. Metrics included change in knowledge, change in intentions, state of behavior change, user experience with BCTs, perceived potential of BCTs, and user engagement with BCTs.

### Design Challenges, Implementation Considerations, and Future Directions

In addition to describing their design process, studies also identified key implementation considerations, design challenges, and future directions for mHealth DBCI design. These have been summarized in Textbox 1. Multimedia Appendix 7 expands on these results in further detail.

**Key design challenges, implementation considerations, and future directions.**

**Design challenges**

- Design process can be time and resource consuming, especially when the design approach is unclear.
- Recruiting and involving representative end users and key stakeholders can be difficult.
- Conflicting ideas can result from integrating behavioral theory, user needs, and stakeholder views.
- The translation of behavior change techniques into actual mobile health (mHealth) digital behavior change intervention (DBCI) features and content can be confusing.
- The design process is time limited and usually may not allow for comprehensive evaluation.
- Integrating the mHealth DBCI into clinical practice can be complex.
- mHealth platforms come with their own technical challenges and limiting factors.

**Implementation considerations**

- Evaluate potential implementation barriers and facilitators during the “testing” step.
- Facilitate early stakeholder buy-in.
- Plan for the integration of the mHealth DBCI into clinical systems.
- Use feasibility criteria throughout the entire design process.
- Use an implementation plan (marketing, dissemination, onboarding, adoption, usage, sustainability).

**Future directions**

- Guidance on the design process for mHealth DBCIs.
- Guidance on how to operationalize behavioral change techniques within mHealth DBCIs in a user-friendly way.
- Guidance on how to meaningfully involve users and stakeholders in the design process.
- Guidance on how to tailor mHealth DBCIs to meet behavioral, personal, and clinical needs.

## Discussion

### Primary Findings

This paper presents a systematic scoping review of 75 papers that described their design process for developing an mHealth DBCI. This review addressed a gap in the literature about how mHealth interventions can be designed to integrate practices from DT and BD. Although the number of mHealth DBCIs seems to be growing, the results highlight substantial heterogeneity in the methods studies used to design them. This scoping review aimed to clarify, map, and synthesize the different methods that can be used to design mHealth DBCIs. A new consolidated approach to mHealth DBCI design is presented—the Behavioral Design Thinking Approach. A large proportion of mHealth DBCIs were designed to facilitate diet- and exercise-related behavior change. Similar findings were noted in a scoping review on DBCI design over the past 2 decades [22]. Nonetheless, the results point to a lack of clarity about how mHealth DBCI content and features were ideated and operationalized. Several studies in this review struggled to translate BCTs into user-friendly mHealth features. Studies also ended up trying to fit several BCTs within the mHealth app, increasing app complexity. To simplify the user experience, several studies noted that personalization may be an important future direction for the field. Tailoring mHealth content to users’ unique behavioral, clinical, and personal needs can help facilitate the delivery of features to support effective engagement [33]. Ensuring that design teams use appropriate questions and metrics to inform personalization will be essential. This further exemplifies the need for the meaningful involvement of patients and the public in mHealth DBCI design. Regarding the meaningful involvement of patients and the public in the mHealth DBCI design process, this review found that patients and the public were most often involved as participants (eg, in user interviews, surveys, testing) rather than as partners on the design team itself. Patients and the public appeared to have minimal direct input on design decision-making throughout the mHealth DBCI design process. Growing calls are being made to prioritize the unique perspectives of patients and the public during design [16,17]. Involving end users in the design process has been suggested to increase the effectiveness, relevance, and appropriateness of mHealth DBCIs [16,17]. Nonetheless, design teams appear to have little guidance on how to meaningfully engage patient and public end users in the design process itself [34-37].

### Theories, Models, and Frameworks Used During Design

Regarding the theories, models, and frameworks that studies used during mHealth DBCI design, design teams appeared to face a vast array of these “tools” from different fields, created for different purposes. Although studies reported benefiting from the structure that these tools offered, a best practice approach for integrating DT and BD insights to develop “effectively engaging” mHealth DBCIs did not appear to exist. This scoping review offers a classification of the types of theories, models, and frameworks that teams can use during mHealth DBCI design and offers a consolidated approach for guiding the design process in general.

### Methods Used to Integrate BD and DT During the mHealth DBCI Design Process

During the integration of DT and BD throughout the mHealth DBCI design, key similarities and differences between the 2 approaches were observed. During the empathizing step, users’ preferences often differed from what was needed to change their behavior. Balancing user-stated preferences with evidence-based behavior change strategies and mHealth platform requirements was a challenge for teams. During the defining step, it was clear that blending DT and BD perspectives resulted in lengthy and complex requirements. Involving users and topic experts to refine these requirements appeared to be a helpful strategy. During ideation, BD relied on evidence-based linkages to ideate content, whereas DT relied on iterative brainstorming and collaborative creation. Studies reconciled these different approaches by starting with BD to develop a list of relevant BCTs and then using DT to creatively integrate BCTs and other requirements into the mHealth DBCI. The prototyping step was the least well described among studies but usually relied on approaches traditional to DT. Studies that brought on their technology partner only at the prototyping stage often had to add additional methods to ensure that BCTs were operationalized as imagined. The testing step was also dominated by DT, as it was challenging to meaningfully test for behavior change outcomes within the time constraints imposed by the design process. Nonetheless, several measures were used by studies to assess the solution’s potential to support behavior change.

### Design Challenges, Implementation Considerations, and Future Directions

Finally, regarding key implementation considerations, design challenges, and future directions, several cross-cutting themes were identified. In addition to ensuring that BD and DT insights were incorporated into the mHealth DBCI design, many teams noted that planning for implementation of the mHealth DBCI was just as important for ensuring success. Using insights from BD and DT may also be relevant in designing an implementation plan for the mHealth DBCI. Implementation planning appears to be particularly complex in this context, as many mHealth DBCIs need to be integrated with existing clinical systems and norms. Regardless of how evidence-based and user-friendly the mHealth DBCI may be, if stakeholder buy-in, system adaptation, and clinical sustainability issues are not taken into consideration, the mHealth DBCIs are likely to fail. Future research must address the lack of guidance design teams have in developing and implementing effective mHealth DBCIs. Particularly, design teams have little guidance on the “tools” (ie, theories, models, and frameworks) they can use, how to meaningfully involve patient and public end users, and how to tailor mHealth DBCI content to meet behavioral, personal, and clinical needs.

### Limitations

The most significant limitation of this scoping review was that the inclusion criteria necessitated that each included paper had to be a full-text primary study that clearly described the mHealth design process. This decision was made to tighten the scope of this review and owing to limited time and resources. Although the systematic database search was not supplemented by a gray literature search or a review of reference lists, the 75 included papers offer a diverse range of insights that meet the research objectives while addressing a prominent gap in the literature. It should be noted that the original scope of this review included a database, reference list, and gray literature search to identify available design frameworks that could be used by design teams to integrate BD and DT insights during mHealth development. Presenting these results alongside the data extracted from 75 primary studies was beyond the scope of this paper. The results of this scoping review are only inclusive of studies written in English; therefore, findings may not be generalizable internationally.

### Implications

The Behavioral Design Thinking Approach offers a way forward in the field of mHealth DBCI design. mHealth design teams may consider using the insights presented in the Behavioral Design Thinking Approach to inform their future work. mHealth design teams may also find it helpful to reflect on the different types of theories, models, and frameworks they can use during the design process, as well as the key challenges they may face along the way. The findings presented in this review may also be relevant to researchers in the fields of behavioral science and design science who are interested in interdisciplinary collaboration. It is reasonable to assume that breaking down silos between these 2 fields may improve the success of mHealth DBCIs. Overall, the main benefits of this research include (1) clarifying what approaches can be used to design mHealth DBCIs, (2) promoting transparency in the choices that studies must make during the mHealth DBCI design process, and (3) enabling future researchers to test what design approaches are the most effective to develop “effectively engaging” mHealth DBCIs.

### Conclusion

The number of mHealth interventions designed to support behavior change is increasing. Integrating best practices from BD and DT may allow for the development of mHealth DBCIs that more effectively engage patients and the public. This paper has helped identify and conceptualize the methods that can be used to integrate BD and DT throughout the mHealth DBCI design process. Raising the standard of mHealth design methods will be essential to ensure confidence in the impact mHealth interventions can have on improving health outcomes. If more mHealth DBCIs are purposefully designed to address effective engagement, it is likely that patients, the public, and health care practitioners will be more confident in adopting mHealth. Given the predicted increase in the demand of mHealth interventions, the time is now to ensure mHealth design methods are appropriately suited to increase effective engagement.

## Appendix

Multimedia Appendix 1 PRISMA-ScR (Preferred Reporting Items for Systematic Reviews and Meta-Analyses Extension for Scoping Reviews) checklist.

Multimedia Appendix 2 Search strategy.

Multimedia Appendix 3 Main characteristics of the studies.

Multimedia Appendix 4 Summary of all the studies included.

Multimedia Appendix 5 Theories, models, and frameworks used in design.

Multimedia Appendix 6 The behavioral design thinking approach.

Multimedia Appendix 7 Design challenges, implementation considerations, and future directions.

## Abbreviations

BCT: behavior change technique
BD: Behavioral Design
DBCI: digital behavior change intervention
DT: Design Thinking
mHealth: mobile health
PRISMA-ScR: Preferred Reporting Items for Systematic Reviews and Meta-Analyses Extension for Scoping Reviews
