# Supplementary material for: Integrating Behavioral Science and Design Thinking to Develop Mobile Health Interventions: Systematic Scoping Review
Source: JMIR Mhealth Uhealth. 2022 Mar 16;10(3):e35799. doi: 10.2196/35799 (PMC8968622; doi:10.2196/35799)
Supplement: Multimedia Appendix 2 [file mhealth_v10i3e35799_app2.doc]

**Multimedia Appendix 2:** **Search strategy**

***Academic Database Search (as completed in MEDLINE)***

**Theme 1: Behavior change**

1. ((behaviou* or behavio*) adj3 (chang* or modif* or interven* or strateg*)).tw,kw.

2. ((behaviou* or behavio*) adj3 (theor* or techni* or model* or principle* or framework* or design* or insight* or economic* or science* or approach* or method*)).tw,kw.

3. (COM-B or BCT or CALO-RE or TDF or BCW or SCT or SDT or TPB or EAST or MINDSPACE).tw,kw.

4. (social cognitive theory or transtheoretical model or self determination theory or motivational interviewing or theory of planned behavior or social learning theory or theory of reasoned action or stages of change or health belief model or theoretical domains framework or behaviour change wheel or personalization theory or social practice theory or nudg* or choice architecture).tw,kw.

**Theme 2: mHealth interventions**

5. Telemedicine/

6. Smartphone/

7. Cell Phones/

8. Text Messaging/

9. Electronic Mail/

10. Computers, Handheld/

11. Mobile Applications/

12. Medical Informatics Applications/

13. Health Information Exchange/

14. (mobile health* or mobile care or mhealth* or m health*).tw,kw.

15. (electronic health* or electronic care or ehealth* or e health*).tw,kw.

16. (digital* adj2 health).tw,kw.

17. (telemedicine or tele medicine or telehealth or tele health or telecare or tele care or telemonitoring or tele monitoring).tw,kw.

18. (mobile adj3 (device* or phone* or telephone* or technolog* or app* or application* or communicat*)).tw,kw.

19. (digital adj3 (device* or phone* or telephone* or technolog* or app* or application* or communicat*)).tw,kw.

20. (electronic adj3 (device* or phone* or telephone* or technolog* or app* or application* or communicat*)).tw,kw.

21. (cell* phone* or cellphone* or smartphone* or smart phone* or iPhone or iPad or android* or blackberr* or computer tablet* or pc tablet* or palmtop computer* or palm top computer* or pda computer*or pocket pc* or pda phone or blackberry or palm pilot* or pilot palm*).tw,kw.

22. (app or apps).tw,kw.

23. (text messag* or electronic mail* or email* or e mail* or short messag* service or sms or multimedia service or multi media service or mms or web messag*).tw,kw.

24. (whatsapp or whats app or instagram or facebook or twitter).tw,kw.

25. (web messag* or instant messag*).tw,kw.

26. ((handheld or hand held) adj computer*).tw,kw.

27. ((handheld or hand held) adj2 console).tw,kw.

28. (elearning or e learning).tw,kw.

29. (social adj2 (media or network*)).tw,kw.

**Theme 3: Design thinking approaches**

30. (user adj2 design).tw,kw.

31. (persuasive adj2 design).tw,kw.

32. gamification.tw,kw.

33. UCD.tw,kw.

34. human cent* design.tw,kw.

35. user cent* design.tw,kw.

36. design thinking.tw,kw.

37. intervention mapping.tw,kw.

38. IDEAS framework.tw,kw.

39. person based approach.tw,kw.

40. participatory design.tw,kw.

41. user driven development.tw,kw.

42. cooperative design.tw,kw.

43. contextual design.tw,kw.

44. empathetic design.tw,kw.

45. activity centred design.tw,kw.

46. (interaction adj2 design).tw,kw.

47. (experience adj2 design).tw,kw.

48. process centred design.tw,kw.

49. (agile adj2 design).tw,kw.

50. (lean adj2 design).tw,kw.

51. design sprint.tw,kw.

52. (waterfall adj2 design).tw,kw.

53. (design science* or design process* or design method* or codesign or co design or codevelop* or co develop* or cocreate or co create* or co produc* or coproduc* or coconstruct* or co construct*).tw,kw.

54. user experience.tw,kw.

55. design requirement*.tw,kw.

56. user requirement*.tw,kw.

57. storyboarding.tw,kw.

58. story mapping.tw,kw.

59. (prototyping or prototype).tw,kw.

60. usability testing.tw,kw.

61. usability test.tw,kw.

62. heuristic eval*.tw,kw.

**Combination of Terms**

63. or/1-4

64. or/5-29

65. or/30-64

63 and 64 and 64

limit to English

***Additional JMIR Search***

(behav* chang* OR behav* insight*  OR behav* theor* ) AND (design thinking OR user centred design  OR user centered design OR human centred design OR human centered design)
