# Supplementary material for: Integrating Behavioral Science and Design Thinking to Develop Mobile Health Interventions: Systematic Scoping Review
Source: JMIR Mhealth Uhealth. 2022 Mar 16;10(3):e35799. doi: 10.2196/35799 (PMC8968622; doi:10.2196/35799)
Supplement: Multimedia Appendix 3 [file mhealth_v10i3e35799_app3.doc]

**Multimedia Appendix 3: Main Characteristics of Included Papers**

| **Main Characteristics of Included Papers (n=75)** | **n (%)** |
| --- | --- |
| **Year of Publication** | |
| 2021 | 2 (2.67) |
| 2020 | 22 (29.33) |
| 2019 | 12 (16.00) |
| 2018 | 15 (20.00) |
| 2017 | 6 (8.00) |
| 2016 | 7 (9.33) |
| 2015 | 4 (5.33) |
| 2014 | 4 (5.33) |
| 2013 | 2 (2.67) |
| 2012 | 1 (1.33) |
|  | |
| **Journal of Publication*** | |
| Journal of Medical Internet Research (JMIR) mHealth and uHealth | 19 (25.33) |
| JMIR Research Protocols | 9 (12.00) |
| JMIR Formative Research | 5 (6.67) |
| JMIR Journal of Medical Internet Research | 4 (5.33) |
| Translational Behavioral Medicine | 4 (5.33) |
| JMIR Publications | 3 (4.00) |
| BioMed Central (BMC) Health Services Research | 2 (2.67) |
| BMC Medical Informatics and Decision Making | 2 (2.67) |
| BMC Public Health | 2 (2.67) |
| JMIR Human Factors | 2 (2.67) |
| PLoS One | 2 (2.67) |
| *All other journals can be viewed in Multimedia Appendix 4 | |
|  | |
| **Location of Publication** | |
| United States | 14 (18.67) |
| United Kingdom | 13 (17.33) |
| Australia | 10 (13.33) |
| Holland | 9 (12.00) |
| Norway | 3 (4.00) |
| New Zealand | 3 (4.00) |
| Palestine | 2 (2.67) |
| China | 2 (2.67) |
| Belgium | 2 (2.67) |
| Portugal | 2 (2.67) |
| Italy | 2 (2.67) |
| Canada | 2 (2.67) |
| Korea | 2 (2.67) |
| South Africa | 2 (2.67) |
| Germany | 2 (2.67) |
| European Union (General) | 2 (2.67) |
| Taiwan | 1 (1.33) |
| Denmark | 1 (1.33) |
| Ireland | 1 (1.33) |
| Bolivia | 1 (1.33) |
| Tajikistan | 1 (1.33) |
| Spain | 1 (1.33) |
| International (General) | 1 (1.33) |
| Switzerland | 1 (1.33) |
|  | |
| **Target Population (and Health Issue)** |  |
| Patients with cardiovascular issues | 7 (9.33) |
| Patients with diabetes | 5 (6.67) |
| Adults with overweight and obesity | 5 (6.67) |
| Adults who smoke | 5 (6.67) |
| Adults with poor physical activity levels | 5 (6.67) |
| Cancer patients and survivors | 5 (6.67) |
| Pregnant women (and men supporting pregnancy) | 4 (5.33) |
| Children with overweight and obesity | 4 (5.33) |
| Adults with alcohol consumption issues | 3 (4.00) |
| Patients with mental illness | 2 (2.67) |
| Patients with periodontal disease | 2 (2.67) |
| Adults (General) | 2 (2.67) |
| Children (General) | 2 (2.67) |
| Adults at risk of cancer | 2 (2.67) |
| Indigenous community members | 2 (2.67) |
| Veterans at risk of metabolic syndrome | 1 (1.33) |
| Patients recovering from surgery | 1 (1.33) |
| Patients with multiple sclerosis | 1 (1.33) |
| Adults with poor diet | 1 (1.33) |
| African-United States adult women | 1 (1.33) |
| Patients with chronic obstructive pulmonary disease (COPD) | 1 (1.33) |
| Patients (General) | 1 (1.33) |
| Adults with hearing aids | 1 (1.33) |
| Patients with human immunodeficiency virus (HIV) (Male) | 1 (1.33) |
| Patients with Asthma | 1 (1.33) |
| Patients with an allogenic stem cell transplant | 1 (1.33) |
| Adults with a recent circumcision | 1 (1.33) |
| Youth drivers | 1 (1.33) |
| Patients with Rheumatoid Arthritis | 1 (1.33) |
| Seniors at risk of fall | 1 (1.33) |
| Adults with Autism Spectrum Disorder | 1 (1.33) |
|  | |
| **Target Health Behavior** | |
| Improved physical activity | 18 (24.00) |
| Improved diet | 17 (22.67) |
| Self-management of disease | 12 (16.00) |
| Preventative health behaviors | 6 (8.00) |
| Adherence to prescribed medication | 5 (6.67) |
| Adherence to a rehabilitation plan | 5 (6.67) |
| Cessation of smoking | 5 (6.67) |
| Self-regulation of weight | 3 (4.00) |
| Self-regulation of alcohol consultation | 3 (4.00) |
| Sexual services uptake/safe sex practices | 3 (4.00) |
| Self-regulation of sleep, sedentary, activity states | 3 (4.00) |
| General healthy lifestyle behaviors | 2 (2.67) |
| Recording medical consultations | 1 (1.33) |
| Proper hearing aid usage | 1 (1.33) |
| Supportive breastfeeding behavior | 1 (1.33) |
|  | |
| **Design Process Duration** | |
| Less than 3 months | 2 (14.29) |
| 3 to less than 6 months | 1 (7.14) |
| 6 to less than 9 months | 3 (21.43) |
| 9 to less than 12 months | 3 (21.43) |
| Longer than 12 months | 5 (35.71) |
