# Supplementary material for: Integrating Behavioral Science and Design Thinking to Develop Mobile Health Interventions: Systematic Scoping Review
Source: JMIR Mhealth Uhealth. 2022 Mar 16;10(3):e35799. doi: 10.2196/35799 (PMC8968622; doi:10.2196/35799)
Supplement: Multimedia Appendix 4 [file mhealth_v10i3e35799_app4.doc]

**Multimedia Appendix 4 – List of all 75 papers included in the analysis**

| **Citation** | **Journal** | **Location** | **Target Population** | **Target Behavior** | **Aim of Study and Design Approach** |
| --- | --- | --- | --- | --- | --- |
| Adu et al, 2020 | Scientific Reports | Australia | Patients with Type 1 or Type 2 diabetes | Self-management of diabetes | This study aimed to describe the development of the My Care Hub mobile application. The design approach was the result of a User Centred Design process and a systematic review of essential factors when developing a diabetes app, which included user involvement, behavior change theory integration, expert consensus, data security considerations, design translation into an app, and usability testing. |
| Antypas et al, 2014 | JMIR Research Protocols | Norway | Patients after cardiac rehabilitation | Adherence to regular physical activity | This study aimed to identify an appropriate theoretical framework, conduct a user needs analysis and combine these to design an effective mobile intervention. The design approach was based on evidence suggesting that using theoretical framework and involving users in the design process could increase intervention effectiveness. |
| Asbjornsen et al, 2020 | JMIR | Norway | Adults who recently lost weight | Weight loss maintenance | This study aimed to present a concept for integrating Persuasive Design principles and Behavior Change Techniques into the design process of eHealth interventions to meet user values and needs. The design approach was based on the Double Diamond Framework and the Center for eHealth Research and Disease Management Roadmap. |
| Athilingam et al, 2018 | Computers, Informatics, Nursing | United States | Patients suffering from heart failure | Self-care during heart failure | This study aimed to describe the six-step Intervention Mapping approach to develop the HeartMapp. The design approach followed the Intervention Mapping approach which comprised of an initial needs assessment, an outline of program objective, the selection of theory-based methods, the translation of objectives into an actual program plan, an adaptation and implementation plan, and an evaluation plan. |
| Bartlett et al, 2020 | JMIR | United Kingdom | Type 2 diabetes patients | Adherence to diabetes medication | This study aimed to develop a text-message intervention that used specific evidence-derived Behavior Change Techniques (BCTs) and was acceptable to people with type 2 diabetes. The design approach was based on ensuring BCT fidelity and to ensure the messages were acceptable to the target population. |
| Beentjes et al, 2016 | BMC Health Services Research | Holland | Patients with severe mental health illnesses | Self-management of mental illness | This study aimed to describe the development of an eHealth application for an Illness Management and Recovery programme. The design approach was based on Intervention Mapping, relying on qualitative evaluations, structured interviews, discussion with a development group, and literature reviews of theoretical models, behavioral change techniques, user studies, and telemedicine interventions. |
| Bogale et al, 2020 | BMC Medical Informatics and Decision Making | Palestine | High risk pregnant women | Timely attendance to antenatal care | This study aimed to describe the planning, development, and evaluation of a theory led intervention using text messages. The design approach utilized a theory-driven process from intervention conceptualization to assessment, guided by the Health Belief Model. |
| Brendryen et al, 2013 | JMIR Research Protocols | Norway | People at risk of alcohol addition | Self-regulation of alcohol consumption | This study aimed to give a systematic and comprehensive description of the treatment rationale of an online alcohol intervention called Balance. The design approach was founded on Intervention Mapping to provide a logical, methodic, step-by-step procedure that helps researchers organize their thoughts as they move from theory and evidence to practice. |
| Buman et al, 2015 | Springer | United States | Veterans at risk of metabolic syndrome | Regulation of 24-hour sleep, sedentary and activity behavior | This study aimed to describe the development and process evaluation of BeWell24, a multicomponent smartphone application that targets behavior change in these interdependent behaviors. The design approach was based on a Community Embedded Iterative Design Framework. |
| Carter et al, 2020 | Stud Health Technol Inform | United States | Patients with psychological stress | Self-management of stress | This study aimed to present a novel theory-driven approach to develop and implement a sustainable mobile application for stress education and management, NewCope. The design approach integrated the Behavior Change Technique Taxonomy and the Patient Engagement Framework to consolidate unique behavior change concepts and user engagement features in a mobile format. |
| Chang et al, 2019 | Plos One | Taiwan | Patients at risk of periodontal disease | Adherence to dental hygiene routine | This study aimed to demonstrate the systematic development process and content of the oral self-care mobile application, OSCA. The design approach was based on the Behavior Change Wheel Framework and comprised of behavioral diagnosis, intervention design, and assessment of OSCA. |
| Chen et al, 2019 | BMC Public Health | China | Patients with coronary heart disease | Adherence to healthy diet and physical activity | This study aimed to systematically design theory-based and evidence-based, individualized, and intelligent interventions. The design approach used the Intervention Mapping framework in order to facilitate a stepwise process where effective evidence-based methods and behavior change theories could be selected. |
| Chen et al, 2020 | JMIR mHealth and uHealth | China | Young adult men who smoke | Cessation of smoking | This study aimed to provide details of the SCAMPI program and its development. The design approach was based on the Behavior Change Wheel and Collaborative Product Development to address behavioral factors identified while also ensuring the design process was user centred. |
| Coumans et al, 2020 | JMIR Research Protocols | Holland | Any population at risk of an unhealthy lifestyle | Improvement of diet and physical activity behaviors | This study aimed to describe the development, design, and evaluation protocol of a web-based computer-tailored intervention, MyLifestyleCoach. The design approach utilized the Intervention Mapping protocol which was used to guide theory and evidence-based decision-making during the design, implementation, and evaluation of this new intervention. |
| Curtis et al, 2015 | JMIR mHealth and uHealth | United Kingdom | Parents of overweight and obese children | Serving appropriately sized food portions | This study aimed to systematically design and develop a theory and evidence-driven, user-centered app. The design approach was guided by The Behavior Change Wheel and User-Centred Design in order to ensure the process was theoretically-based and also met user needs. |
| Dalum et al, 2016 | Health Promotion Practice | Denmark | Adults who wished to quit smoking | Cessation of smoking | This study aimed to determine whether Intervention Mapping was a suitable strategy for the development of an Internet- and text message-based smoking cessation intervention. The design approach was based on Intervention Mapping to link behavioral goals, theoretical methods, and practical strategies and materials. |
| den Bakker et al, 2019 | Journal of Medical Internet Research | Holland | Patients who have undergone a surgery | Self-management of at-home recovery | This study aimed to develop the "ikherstel" eHealth intervention. The design approach was based on Intervention Mapping which was deemed a suitable systematic and scientifically accepted method for the development and implementation of a wide range of eHealth interventions. |
| DeSmet et al, 2019 | JMIR mHealth and uHealth | Belgium | Adults who want healthier 24-hour movement | Self-monitoring of 24-hour sleep, sedentary and activity behavior | This study aimed to examine adult users’ preferences for techniques and features in mobile apps for 24-hour movement behaviors. The design approach was based on user centred design to explore and integrate user preferences for behavior change techniques and engagement features. |
| Direito et al, 2018 | Health Education & Behavior | New Zealand | Healthy but insufficiently active adults | Increased physical activity and decreased sedentary behavior | This study aimed to describe the application of two frameworks to assist the development of an adaptive evidence-based smartphone-delivered intervention. The design approach was based on Intervention Mapping (to identify the determinants influencing uptake of physical activity and behavior change techniques) and the Behavioral Intervention Technology model (to translate and operationalize the BCTs and its modes of delivery). |
| Duff et al, 2018 | JMIR Formative Research | Ireland | Adults with cardiovascular disease | Adherence to cardiac rehabilitation programming | This study aimed to develop a behavior change, theoretically informed exercise rehabilitation mobile app for adults with CVD, MedFit. The design approach relied on the mHealth Development and Evaluation Framework as well as a user centred approach in order to develop the app based on theory, usability testing and content design. |
| Edwards et al, 2018 | JMIR Serious Games | United Kingdom | Adult smokers | Cessation of smoking | This study aimed to present a series of steps undertaken during the development of Cigbreak, a gamified smoking cessation health app. The design approach followed principles of Agile Development which allows for prototypes to be developed rapidly and systematically modified according to user feedback. |
| Felix et al, 2019 | Frontiers in Pharmacology | Portugal | Seniors with Type 2 Diabetes | Adherence to medication | This study aimed to describe the development of a theory and evidence-based complex intervention to improve adherence to oral antidiabetics. The design approach was based on The Behavior Change Wheel (to develop of theoretical understanding of the behavior change process) and the MRC Framework for Complex Interventions (to guide methodological decision making). |
| Fuemeller et al, 2020 | Games for Health Journal | United States | Survivors of childhood cancer | Adherence to healthy diet and physical activity | This study aimed to develop an app-based intervention, using game design characteristics, theory-based behavioral strategies, and assistance from a health coach to motivate health behavior change for adolescent survivors of childhood cancer. The design approach was guided by a theoretical framework specifically created to guide the app development process. |
| Gabrielli et al, 2017 | JMIR mHealth and uHealth | Italy | Overweight children, and their parents | Adherence to healthy eating changes | This study aimed to describe the design and development of the TreC-LifeStyle nutrition education app. The design approach was based on a multidisciplinary User Centred Design approach informed by theoretical-empirical knowledge to ensure future mHealth intervention adoption. |
| Garnett et al, 2018 | Translational Behavioral Medicine | United Kingdom | Individuals who consume alcohol excessively | Reduction of alcohol consumption | This study aimed to describe the development of a smartphone app to help individuals who drink excessively to reduce their alcohol consumption. The design approach was guided by the MRC Framework for Complex Interventions (to ensure the use of theory and evidence in the selection of intervention components) and the Person Based Approach (to translate intervention components into appropriate app modules). |
| Giunti et al, 2018 | JMIR mHealth and uHealth | European Union | Young adults with multiple sclerosis | Self-management of fatigue | This study aimed to describe the design and evaluation process of a gamified mHealth solution for behavioral change in persons with MS. The design approach was based on User Centred Design to ensure the solution was more likely to meet end users’ needs and expectations. |
| Hales et al, 2016 | JMIR Human Factors | United States | Overweight and obese adults | Self-management of healthy diet and physical activity | This study aimed to develop, refine, and pilot test the Social Pounds Off Digitally (POD) Android app for personalized health monitoring and interaction. The design approach consisted of programming the selection of messages targeting behavioral theory constructs in order to re-engage infrequent users. |
| Hawley-Hague et al, 2020 | JMIR mHealth and uHealth | Italy | Senior patients at risk of falls | Adherence to exercise program for falls reduction | This study aimed to develop motivational smartphone apps co-designed with health professionals and older adults to support patients to perform exercise proven to aid fall reduction. The design approach was guided by the MRC Framework for Complex Interventions and a Human Centred Design Approach to include a range of interacting behavior change components and adaptation to user needs. |
| Hebden et al, 2012 | JMIR Research Protocols | Australia | Young adults at risk of overweight or obesity | Adherence to healthy lifestyle changes (diet and physical activity related) | This study aimed to describe the process of developing apps aimed at modifying key lifestyle behaviors associated with weight gain in young adulthood. The design approach involved deciding on specifications, selecting a platform, designing the intervention, and testing the prototype. |
| Hendrie et al, 2019 | JMIR Formative Research | Australia | Adults who want to improve their diets | Adherence to having three different vegetables for dinner | This study aimed to describe the iterative process of developing a theory-based smartphone app, VegEze. The design approach was based on the Integrate, Design, Assess, and Share Framework (to integrate behavioral theory, design thinking, user centred design rigorous evaluation and dissemination) and the Behavior Change Wheel (to guide a deeper understanding of behavior change). |
| Hooglugt et al, 2020 | JMIR Publications | Holland | Anyone who wants to improve their physical activity | Self-regulation of physical activity | This study aimed to outline a method for web-based or mobile intervention development that allows exploration of integrating behavior change theory into the design process. The design approach was based on the Research through Design Process, in which design activities were carried out as part of the knowledge generating process. |
| Joseph et al, 2020 | JMIR Publications | United States | African- United States n adult women | Adherence to regular physical activity | This study aimed to describe the development and initial usability testing of Smart Walk, a culturally relevant social cognitive theory-based smartphone intervention. The design approach was a 5-phase process that involved user focus groups, social cognitive theory and cultural tailoring, multimedia material integration, iterative prototype development and preliminary user testing. |
| Kazemi et al, 2018 | Addiction Research & Theory | United States | College students with risky alcohol consumption | Self-monitoring of hazardous alcohol drinking | This study aimed to develop a self-monitoring app, REMIT, informed by current behavioral theories to reduce hazardous drinking among college students. The design approach was informed by the Integrate, Design, Assess, and Share Framework to understand users, determine the target behavior, ground in behavioral theory, creative intervention strategies and develop a prototype. |
| Kim et al, 2020 | PLoS ONE | United States and Korea | Adults with autism spectrum disorder | Adherence to regular physical activity | This study aimed to describe the iterative design process of PuzzleWalk, a gamified, physical activity promoting mobile app for adults with autism spectrum disorder, reflecting on lessons learned across four User Centred Design phases. The design approach was based on User Centred Design in order to focus on users and their requirements in each step of the design process. |
| Korpershoek et al, 2020 | Journal of Medical Internet Research | Holland | Patients with COPD | Self-management of COPD exasperations | This study aimed to describe in detail the User Centred Design process of an evidence-driven mHealth intervention to enhance exacerbation-related self-management in patients with COPD. The design approach relied on the User Centred Design process, MRC Framework for Complex Interventions, and the Behavior Change wheel. |
| Lee et al, 2013 | JMIR Research Protocols | Korea | Cancer survivors who need to manage their weight | Adherence to exercise and diet program | This study aimed to develop a Web-based self-management diet and exercise intervention program with the aid of the transtheoretical model. The design approach was a 5-phase system development life-cycle (SDLC) method. |
| Lipson-Smith et al, 2019 | JMIR Formative Research | Australia | Patients who want to record their medical consultations | Recording of medication consultations | This study aimed to use a co-design process to develop a consultation audio-recording mobile app called SecondEars. The design approach was based on Experience Based Co-Design to ensure SecondEars was a patient-identified solution while meeting the implementation requirements identified by clinicians and administrators. |
| Maidment et al, 2020 | International Journal of Audiology | United Kingdom | First-time hearing aid users | Proper hearing aid usage | This study aimed to describe the development of a novel mobile health educational intervention, called m2Hear, designed for first-time hearing aid users based on previously developed educational multimedia videos. The design approach was based on a User-Centred Participatory Design Approach to improve accessibility. |
| Mann et al, 2014 | JMIR mHealth and uHealth | United States | Primary care patients with hypertension | Increased physical activity and healthy diet changes | This study aimed to develop a behavioral hypertension reduction mHealth system inspired by a lifestyle modification program, DASH. The design approach appeared to pull from perspectives of User Centred Design and was used to reflect on key lessons learned about mHealth behavior change design. |
| Marie Brown et al, 2020 | JMIR Formative Research | Canada | Children between the age of 9-12 | Increased knowledge about food and nutrition | This study aimed to describe the iterative development and user testing of Foodbot Factory, a novel nutrition education gamified app for children to use at home or in the classroom. The design approach was founded on the Obesity-Related Behavioral Intervention Trials (ORBIT) Model, which emphases developing behavioral interventions and is highly complementary to frameworks used by game developers and to chronic disease prevention programming. |
| Mathenjwa et al, 2020 | JMIR mHealth and uHealth | South Africa | Men with HIV | Anti-retroviral usage and adherence | This study aimed to deliver a usable and engaging tablet-based app, called EPIC-HIV 2 (Empowering People through Informed Choices for HIV 2), to support men in making informed decisions about engaging with HIV care. The design approach was guided by the Person-Based Approach and Human Computer Interaction Techniques to integrate evidence-based content and design to ensure the app was relatable and engaging for users. |
| McCarthy et al, 2018 | BMC Public Health | Tajikistan, Bolivia, and Palestine | Women with high risk of unintended pregnancy | Increased contraceptive choice | This study aimed to describe the development of a contraceptive behavioral intervention delivered by a mobile phone to address an unmet need for contraception. The design approach was guided by Intervention Mapping to systematically develop the health behavior change intervention. |
| Monteiro-Guerra et al, 2020 | JMIR mHealth and uHealth | Spain | Survivors of breast cancer | Adherence to physical activity program | This study aimed to execute the design process and early prototype evaluation for a personalized physical activity coaching app for breast cancer survivors. The design approach was guided by User Centred Design in order to focus on the users and their needs, which would help ensure the usefulness of the technology. |
| Morrison et al, 2015 | BMC Medical Informatics and Decision Making | United Kingdom | Adults with asthma | Self-management of asthma | This study aimed to develop an evidence based, theory informed, online resource to support self-management in adults with asthma, called ‘Living well with Asthma’, as part of the RAISIN (Randomized Trial of an Asthma Internet Self-Management Intervention) study. The design approach was based on the MRC Framework for Complex Interventions which recommends that intervention development be systematic, include evidence, be theory based and incorporate feasibility/ user testing. |
| Mueller et al, 2019 | British Journal of Health Psychology | United Kingdom | Adults with symptoms of lung cancer | Early help-seeking behavior | This study aimed to detail the development method used to produce an online, tailored, theory-based, user-centred, intervention to encourage help-seeking behavior for lung cancer symptoms. The design approach was structured around the Person-Based Approach, which drew on user interviews, previous research, patient involvement work, think aloud evaluation and a guiding principles document. |
| Mummah et al, 2016 | The International Journal of Behavioral Nutrition and Physical Activity | United States | Overweight adults | Increased vegetable consumption | This study aimed to iteratively develop a theory-driven mobile app, *Vegethon*, to increase vegetable consumption. The design approach was based on the Integrate, Design, Assess, and Share Framework, which provides a comprehensive step-by-step process to mHealth intervention development |
| Nelson et al, 2016 | JMIR Human Factors | United States | Adults with Type 2 Diabetes | Adherence to medication | This study aimed to develop Rapid Education/Encouragement And Communications for Health (REACH), a tailored, text messaging intervention to support the self-care adherence of disadvantaged patients with T2DM. The design approach was structured on a empirical and theory-based approach, findings from a previous intervention and the experience of the research team. |
| Newby et al, 2017 | HIV Prevention & Sexual Health | United Kingdom | Young people (aged 13 to 19) | Uptake of sexual health services | This study aimed to develop and implement an intervention delivered via a web app and website to increase the uptake of sexual health services by young people. The design approach was informed by Intervention Mapping (which provides structured and detailed planning across a series of sequential and iterative steps) and the Behavior Change Wheel (to select suitable behavior change techniques). |
| Partridge et al, 2019 | International Journal of Environmental Research and Public Health | Australia | Adolescents interested in weight loss | Self-management of weight | This study aimed to co-design a bank of text messages that are evidence-based, acceptable, and engaging for adolescents. The design approach was based on an iterative mixed methods process with three phases following a Co-Design Approach. |
| Peleg et al, 2018 | Journal of Medical Systems | International | Patients with Atrial Fibrillation | Adherence to therapy | This study aimed to describe the development of an evidence based clinical decision support system that delivers personalized reminders and recommendations to patients, helping to achieve higher therapy compliance. The design approach follows the Integrate, Design, Assess, and Share (IDEAS) Framework because of its focus on translating behavior theories into conceptual mHealth modalities. |
| Potzel et al, 2021 | JMIR mHealth and uHealth | Germany | Mothers with Gestational Diabetes | Change in cardiometabolic risk behaviors | This study aimed to systematically plan and develop a theory- and evidence – based mHealth intervention to change cardiometabolic risk behaviors in women. The design approach was based on Intervention Mapping to structure the systematic planning and development |
| Puijk-Hekman et al, 2017 | JMIR Research Protocols | Holland | Patients with cardiovascular disease | Self-management of disease | This study aimed to describe the development of "Vascular View," a comprehensive, multi-component, tailored, Web-based, self-management support program for patients with CVD. The design approach was based on Intervention Mapping to develop a theoretically based behavior change intervention. |
| Quintiliani et al, 2019 | Clinical Correspondence | United States | Breast cancer survivors | Improved physical acidity and health diet | This study aimed to examine the perceptions of evidence-based features breast cancer survivors would find important in a future mHealth intervention. The design approach was based on a systematic review of behavior change techniques (BCTs), a PowerPoint presentation of potential BCT incorporation, user discussions, user surveys and prototype usability feedback. |
| Reidy et al, 2020 | JMIR Publications | United Kingdom | Patients with Type 1 Diabetes | Self-management of diabetes | This study aimed to evaluate the perspectives and experiences of people with T1D and health care professionals to determine what behavior change strategies would be required for a web-based social network intervention. The design approach was based on the Behavior Change Wheel, which included a behavioral diagnosis, intervention strategy selection, and selection of specific behavior change techniques. |
| Ribaut et al, 2020 | BMC Health Services Research | Switzerland | Allogenic stem cell transplant (alloSCT) patients | Adherence to medication | This study aimed to report the theory-driven development of an intervention module to support medication adherence as the first step to digitation of the SMILe project. The design approach was based on the Behavior Change Wheel, which was used to rigorously develop and implement the behavior change intervention. |
| Rodrigues et al, 2017 | JMIR Research Protocols | Portugal and United Kingdom | Patients at risk of melanoma | Adherence to sun protection behaviors | This study aimed to describe and appraise the process of systematically developing a smartphone intervention (mISkin app) to promote sun protection during the holidays. The design approach incorporates theory and evidence-based approaches outlined by the MRC Framework and engaging user perspectives throughout the development process. |
| Rohde et al, 2019 | JMIR mHealth and uHealth | Germany | Young Adults from disadvantaged backgrounds | Improved dietary and drinking habits | This study aimed to systematically design a theory-driven and target-group adapted dietary mobile app concept to promote healthy eating habits. The design approach was guided by the Behavior Change Wheel, which involved specifying the target behavior, barriers and facilitators to behavior change, behavior change techniques, app functionalities and features, and experts interview results. |
| Ross et al, 2019 | Patient Preference and Adherence | Australia | Cancer chemotherapy patients | Medication adherence | This study aimed to describe the theoretical, evidence-based, and consumer-informed development of a smartphone self-management program to support adherence to oral chemotherapy. The design approach was based on the Behavioral Intervention Technology (BIT) Model (to guide the integration of behavior and technological aspects of the intervention) and Whittaker et al’s Development and Evaluation Framework for mHealth Interventions (to guide best practices for mHealth design). |
| Sakakibara et al, 2017 | Archives of Physical Medicine and Rehabilitation | Canada | Patients who have had a stroke | Adherence to healthy lifestyle changes | This study aimed to describe the systematic development of the Stroke Coach, a theory- and evidence-based intervention to improve control of lifestyle behavior risk factors in patients with stroke. The design approach was informed by Intervention Mapping, which provided a systematic process for intervention development to integrate theory and evidence. |
| Scheerman et al, 2018 | JMIR mHealth and uHealth | Holland | Patients with dental caries | Adherence to oral health behavior | This study aimed to describe the systematic development and content of the WhiteTeeth app. The design approach was based on Intervention Mapping, which helped plan and develop the theory- and evidence- based health promotion program. |
| Shoneye et al, 2020 | JMIR mHealth and uHealth | Australia | Overweight and obese adults | Self-monitoring of diet and physical activity | This study aimed to build a tailored intervention using mobile technology to improve diet and physical activity behaviors, with the objective to identify behavior change techniques and preferences for digital intervention features. The design approach was based on the Person-Based Approach (to allow for an iterative and multidisciplinary approach that includes qualitative investigation with the user) and the Behavioral Intervention Technology (BIT) Framework (to identify the technology and procedures appropriate for delivering behavior change techniques). |
| Simons et al, 2018 | JMIR mHealth and uHealth | Belgium | Young adults with low physical activity | Self-regulation of physical activity | This study aimed to describe the development, usability, acceptability, and feasibility of a new theory- and evidence-based smartphone app to promote an active lifestyle in lower-educated working young adults. The design approach was based on Intervention Mapping and Whittaker et al’s Development and Evaluation Framework for mHealth Interventions. |
| Sittig et al, 2020 | JMIR mHealth and uHealth | United States | Adults with Type 2 diabetes | Self-management of diabetes | This study aimed to determine the impact of an mHealth app, capability, that incorporated theory-driven trigger messages. The design approach was informed by User Centred Design to ensure the design was reflective of key stakeholder needs. |
| Sporrel et al, 2021 | frontiers in Public Health | Holland | Adults who lived close to a municipal parks | Increased physical activity | This study aimed to describe the design and development of the Playful data-driven Active Urban Living (PAUL): a personalized physical activity application based on different development frameworks for mHealth. The design approach was informed by the Behavior Change Wheel (to gain a deeper understanding of how to facilitate behavior change), the Integrate, Design, Assess, and Share (IDEAS) Framework (to incorporate the needs of the end user throughout design) and the Behavioral Intervention Technology (BIT) Model (to guide the practical implementation of persuasive strategies in the application interface). |
| Stephenson et al, 2020 | Digital Health | United Kingdom | Desk-based office workers | Self-regulation of sedentary behavior | This study aimed to describe the iterative development of a theory-based, digital behavior change intervention to reduce occupational sedentary behavior. The design approach was based on the Behavior Change Wheel (to guide the selection of intervention components), User Centred Design (to involve users throughout design) and the MRC Framework for Complex Intervention (to guide the development process). |
| Toefy et al, 2016 | JMIR mHealth and uHealth | South Africa | Recently circumcised men | Safe sex behavior | This study aimed to develop a participative, theory-based, mobile phone, audio messaging intervention attractive to recently circumcised men at voluntary medical male circumcision (VMMC) clinics in the Cape Town. The design approach was based on a staggered qualitative methodology that considered several theoretical frameworks at different points in the intervention. |
| Tonkin et al, 2017 | JMIR mHealth and uHealth | Australia | Remote Indigenous Australians | Reduction of sugar sweetened beverages | This study aimed to consult remote Indigenous community members to inform the content of a smartphone app that can be used to monitor and reduce sugar-sweetened beverage intake. The design approach was designed based on findings from a scoping review tailored to the Indigenous setting. It involved a formative research phase (to inform app features, content, and structure) and preliminary user testing. |
| van Agteren et al, 2018 | Translational Behavorial Medicine | Australia | Smokers who would like to stop smoking | Adherence to smoking cessation intervention | This study aimed to describe the development of a smoking cessation smartphone application developed using evidence-based principles. The design approach was informed by Intervention Mapping to emphasise taking environmental determinants, behaviors, and outcomes into account to address behavior change. |
| Verbiest et al, 2019 | Translational Behavioral Medicine | New Zealand | Indigenous obese adults in New Zealand | Improved diet and physical activity behavior | This study aimed to provide an overview of the codesign methods and processes and describe how these were used to inform and build a theory-driven approach to the selection of behavioral determinants and change techniques. The design approach was informed by a partnership between Māori and Pasifika partners and an academic research team and was based on a Co-Design Framework. |
| Walsh et al, 2018 | Translational Behavioral Medicine | United Kingdom and the European Union | Patients with cardiovascular diseases | Adherence to cardiac rehabilitation programming | This study aimed to develop the PATHway intervention (physical activity toward health) for the self-management of cardiovascular disease. The design approach was informed by the Behavior Change Wheel (to develop behavior change interventions) and the Behavioral Intervention Technology (BIT) Model (to facilitate the translation of behavioral components into technological features). |
| Warren et al, 2018 | JMIR Formative Research | New Zealand | Youth drivers aged 16 -24 | Increased safe driving behaviors | This study aimed to apply behavioral change principles to design and develop a smartphone-based intervention with the aim of helping youth drivers to develop and hone safe driving skills. The design approach was an iterative process that involved reviewing the behavior change literature, identifying fundamental design principles, stakeholder engagement and reviewing technology adaptation considerations. |
| Waterlander et al, 2014 | JMIR mHealth and uHealth | United Kingdom | Adults who want to lose weight | Self-management of weight | This study aimed to develop an evidence based mHealth weight management program (Horizon) using formative research and a structured content development process. The design approach used mixed methods research involving expert input on effective programming and behavior change theory, and target user engagement. |
| Wen et al, 2014 | JMIR Research Protocols | United States | Woman at risk of smoking relapse after childbirth | Prevention of smoking relapses | This study aimed to describe the development of a social-cognitive theory-based and evidence-guided text messaging intervention for preventing postpartum smoking relapse among inner city women. The design approach was guided by User Centred Design to follow a series of iterative steps to ensure content was understandable and evidence based. |
| White et al, 2015 | JMIR mHealth and uHealth | Australia | Men who have partners who are pregnant | Supportive breastfeeding behaviors | This study aimed to use previous research, formative evaluation, and behavior change theory to develop the first evidence-based breastfeeding app targeted at men. The design approach was founded on a multidisciplinary team approach and a formative research process. |
| Zuidema et al, 2015 | JMIR Research Protocols | Holland | Adolescent patients with Rheumatoid Arthritis | Self-management of Rheumatoid Arthritis | This study aimed to develop an online, computer-tailored, self-management program integrated with the nursing care. The design approach was informed by Intervention Mapping to ensure that the development was focused on most important determinants of behavior. |

**References**

[1] Adu, M. D., Malabu, U. H., Malau-Aduli, A. E. O., & Malau-Aduli, B. S. (2020). The development of My Care Hub mobile-phone app to support self-management in Australians with type 1 or type 2 diabetes. *Scientific Reports*, *10*(1), 1–10.

[2] Antypas, K., & Wangberg, S. C. (2014). Combining users’ needs with health behavior models in designing an internet-and mobile-based intervention for physical activity in cardiac rehabilitation. *JMIR Research Protocols*, *3*(1), e4.

[3] Asbjørnsen, R. A., Wentzel, J., Smedsrød, M. L., Hjelmesæth, J., Clark, M. M., Nes, L. S., & Van Gemert-Pijnen, J. E. W. C. (2020). Identifying persuasive design principles and behavior change techniques supporting end user values and needs in ehealth interventions for long-term weight loss maintenance: qualitative study. *Journal of Medical Internet Research*, *22*(11), e22598.

[4] Athilingam, P., Clochesy, J. M., & Labrador, M. A. (2018). Intervention mapping approach in the design of an interactive mobile health application to improve self-care in heart failure. *CIN: Computers, Informatics, Nursing*, *36*(2), 90–97.

[5] Bartlett, Y. K., Farmer, A., Rea, R., & French, D. P. (2020). Use of brief messages based on behavior change techniques to encourage medication adherence in people with type 2 diabetes: developmental studies. *Journal of Medical Internet Research*, *22*(5), e15989.

[6] Beentjes, T. A. A., van Gaal, B. G. I., Goossens, P. J. J., & Schoonhoven, L. (2015). Development of an e-supported illness management and recovery programme for consumers with severe mental illness using intervention mapping, and design of an early cluster randomized controlled trial. *BMC Health Services Research*, *16*(1), 1–9.

[7] Bogale, B., Mørkrid, K., O’Donnell, B., Ghanem, B., Ward, I. A., Khader, K. A., Isbeih, M., Frost, M., Baniode, M., & Hijaz, T. (2020). Development of a targeted client communication intervention to women using an electronic maternal and child health registry: a qualitative study. *BMC Medical Informatics and Decision Making*, *20*(1), 1–12.

[8] Brendryen, H., Johansen, A. B., Nesvåg, S., Kok, G., & Duckert, F. (2013). Constructing a theory-and evidence-based treatment rationale for complex eHealth interventions: Development of an online alcohol intervention using an intervention mapping approach. *JMIR Research Protocols*, *2*(1), e6.

[9] Brown, J. M., Savaglio, R., Watson, G., Kaplansky, A., LeSage, A., Hughes, J., Kapralos, B., & Arcand, J. (2020). Optimizing child nutrition education with the Foodbot Factory mobile health app: formative evaluation and analysis. *JMIR Formative Research*, *4*(4), e15534.

[10] Buman, M. P., Epstein, D. R., Gutierrez, M., Herb, C., Hollingshead, K., Huberty, J. L., Hekler, E. B., Vega-López, S., Ohri-Vachaspati, P., & Hekler, A. C. (2016). BeWell24: development and process evaluation of a smartphone “app” to improve sleep, sedentary, and active behaviors in US Veterans with increased metabolic risk. *Translational Behavioral Medicine*, *6*(3), 438–448.

[11] Carter, L., Rogith, D., Franklin, A., & Myneni, S. (2019). NewCope: A Theory-Linked Mobile Application for Stress Education and Management. *Studies in Health Technology and Informatics*, *264*, 1150.

[12] Chang, W.-J., Lo, S.-Y., Kuo, C.-L., Wang, Y.-L., & Hsiao, H.-C. (2019). Development of an intervention tool for precision oral self-care: Personalized and evidence-based practice for patients with periodontal disease. *PloS One*, *14*(11), e0225453.

[13] Chen, J., Ho, E., Jiang, Y., Whittaker, R., Yang, T., & Bullen, C. (2020). Mobile Social Network–Based Smoking Cessation Intervention for Chinese Male Smokers: Pilot Randomized Controlled Trial. *JMIR MHealth and UHealth*, *8*(10), e17522.

[14] Chen, Y., Wu, F., Wu, Y., Li, J., Yue, P., Deng, Y., Lamb, K. V, Fong, S., Liu, Y., & Zhang, Y. (2019). Development of interventions for an intelligent and individualized mobile health care system to promote healthy diet and physical activity: using an intervention mapping framework. *BMC Public Health*, *19*(1), 1–16.

[15] Coumans, J. M. J., Bolman, C. A. W., Friederichs, S. A. H., Oenema, A., & Lechner, L. (2020). Development and testing of a personalized web-based diet and physical activity intervention based on motivational interviewing and the self-determination theory: protocol for the mylifestylecoach randomized controlled trial. *JMIR Research Protocols*, *9*(2), e14491.

[16] Curtis, K. E., Lahiri, S., & Brown, K. E. (2015). Targeting parents for childhood weight management: development of a theory-driven and user-centered healthy eating app. *JMIR MHealth and UHealth*, *3*(2), e3857.

[17] Dalum, P., Brandt, C. L., Skov-Ettrup, L., Tolstrup, J., & Kok, G. (2016). The systematic development of an internet-based smoking cessation intervention for adults. *Health Promotion Practice*, *17*(4), 490–500.

[18] den Bakker, C. M., Schaafsma, F. G., van der Meij, E., Meijerink, W. J. H. J., van den Heuvel, B., Baan, A. H., Davids, P. H. P., Scholten, P. C., van der Meij, S., & van Baal, W. M. (2019). Electronic health program to empower patients in returning to normal activities after general surgical and gynecological procedures: Intervention mapping as a useful method for further development. *Journal of Medical Internet Research*, *21*(2), e9938.

[19] DeSmet, A., De Bourdeaudhuij, I., Chastin, S., Crombez, G., Maddison, R., & Cardon, G. (2019). Adults’ preferences for behavior change techniques and engagement features in a mobile app to promote 24-hour movement behaviors: Cross-sectional survey study. *JMIR MHealth and UHealth*, *7*(12), e15707.

[20] Direito, A., Walsh, D., Hinbarji, M., Albatal, R., Tooley, M., Whittaker, R., & Maddison, R. (2018). Using the intervention mapping and behavioral intervention technology frameworks: development of an mHealth intervention for physical activity and sedentary behavior change. *Health Education & Behavior*, *45*(3), 331–348.

[21] Duff, O., Walsh, D., Malone, S., McDermott, L., Furlong, B., O’Connor, N., Moran, K., & Woods, C. (2018). MedFit app, a behavior-changing, theoretically informed mobile app for patient self-management of cardiovascular disease: user-centered development. *JMIR Formative Research*, *2*(1), e9550.

[22] Edwards, E. A., Caton, H., Lumsden, J., Rivas, C., Steed, L., Pirunsarn, Y., Jumbe, S., Newby, C., Shenvi, A., & Mazumdar, S. (2018). Creating a theoretically grounded, gamified health app: Lessons from developing the Cigbreak smoking cessation mobile phone game. *JMIR Serious Games*, *6*(4), e10252.

[23] Engelen, M. M., van Dulmen, S., Puijk-Hekman, S., Vermeulen, H., Nijhuis-van der Sanden, M. W., Bredie, S. J., & van Gaal, B. G. (2020). Evaluation of a Web-Based Self-Management Program for Patients With Cardiovascular Disease: Explorative Randomized Controlled Trial. *Journal of Medical Internet Research*, *22*(7), e17422. https://doi.org/https://dx.doi.org/10.2196/17422

[24] Félix, I. B., Guerreiro, M. P., Cavaco, A., Cláudio, A. P., Mendes, A., Balsa, J., Carmo, M. B., Pimenta, N., & Henriques, A. (2019). Development of a complex intervention to improve adherence to antidiabetic medication in older people using an anthropomorphic virtual assistant software. *Frontiers in Pharmacology*, *10*, 680.

[25] Fuemmeler, B. F., Holzwarth, E., Sheng, Y., Do, E. K., Miller, C. A., Blatt, J., Rosoff, P. M., & Østbye, T. (2020). Mila blooms: a mobile phone application and behavioral intervention for promoting physical activity and a healthy diet among adolescent survivors of childhood cancer. *Games for Health Journal*, *9*(4), 279–289.

[26] Gabrielli, S., Dianti, M., Maimone, R., Betta, M., Filippi, L., Ghezzi, M., & Forti, S. (2017). Design of a mobile app for nutrition education (TreC-LifeStyle) and formative evaluation with families of overweight children. *JMIR MHealth and UHealth*, *5*(4), e7080.

[27] Garnett, C., Crane, D., West, R., Brown, J., & Michie, S. (2019). The development of Drink Less: an alcohol reduction smartphone app for excessive drinkers. *Translational Behavioral Medicine*, *9*(2), 296–307.

[28] Giunti, G., Mylonopoulou, V., & Romero, O. R. (2018). More stamina, a gamified mhealth solution for persons with multiple sclerosis: research through design. *JMIR MHealth and UHealth*, *6*(3), e9437.

[29] Hales, S., Turner-McGrievy, G., Fahim, A., Freix, A., Wilcox, S., Davis, R. E., Huhns, M., & Valafar, H. (2016). A mixed-methods approach to the development, refinement, and pilot testing of social networks for improving healthy behaviors. *JMIR Human Factors*, *3*(1), e4512.

[30] Hawley-Hague, H., Tacconi, C., Mellone, S., Martinez, E., Ford, C., Chiari, L., Helbostad, J., & Todd, C. (2020). Smartphone apps to support falls rehabilitation exercise: app development and usability and acceptability study. *JMIR MHealth and UHealth*, *8*(9), e15460.

[31] Hebden, L., Cook, A., Van Der Ploeg, H. P., & Allman-Farinelli, M. (2012). Development of smartphone applications for nutrition and physical activity behavior change. *JMIR Research Protocols*, *1*(2), e2205.

[32] Hendrie, G. A., James-Martin, G., Williams, G., Brindal, E., Whyte, B., & Crook, A. (2019). The development of VegEze: smartphone app to increase vegetable consumption in Australian adults. *JMIR Formative Research*, *3*(1), e10731.

[33] Hooglugt, F., & Ludden, G. D. S. (2020). A mobile app adopting an identity focus to promote physical activity (MoveDaily): iterative design study. *JMIR MHealth and UHealth*, *8*(6), e16720.

[34] Joseph, R. P., Keller, C., Vega-López, S., Adams, M. A., English, R., Hollingshead, K., Hooker, S. P., Todd, M., Gaesser, G. A., & Ainsworth, B. E. (2020). A culturally relevant smartphone-delivered physical activity intervention for African American women: Development and initial usability tests of smart walk. *JMIR MHealth and UHealth*, *8*(3), e15346.

[35] Kazemi, D. M., Borsari, B., Levine, M. J., Lamberson, K. A., & Dooley, B. (2018). REMIT: Development of a mHealth theory-based intervention to decrease heavy episodic drinking among college students. *Addiction Research & Theory*, *26*(5), 377–385. https://doi.org/https://dx.doi.org/10.1080/16066359.2017.1420783

[36] Kim, B., Lee, D., Min, A., Paik, S., Frey, G., Bellini, S., Han, K., & Shih, P. C. (2020). PuzzleWalk: A theory-driven iterative design inquiry of a mobile game for promoting physical activity in adults with autism spectrum disorder. *Plos One*, *15*(9), e0237966.

[37] Korpershoek, Y. J. G., Hermsen, S., Schoonhoven, L., Schuurmans, M. J., & Trappenburg, J. C. A. (2020). User-centered design of a mobile health intervention to enhance exacerbation-related self-management in patients with chronic obstructive pulmonary disease (copilot): mixed methods study. *Journal of Medical Internet Research*, *22*(6), e15449.

[38] Lee, M. K., Park, H.-A., Yun, Y. H., & Chang, Y. J. (2013). Development and formative evaluation of a web-based self-management exercise and diet intervention program with tailored motivation and action planning for cancer survivors. *JMIR Research Protocols*, *2*(1), e2331.

[39] Lipson-Smith, R., White, F., White, A., Serong, L., Cooper, G., Price-Bell, G., & Hyatt, A. (2019). Co-design of a consultation audio-recording mobile app for people with cancer: the SecondEars app. *JMIR Formative Research*, *3*(1), e11111.

[40] Maidment, D. W., Coulson, N. S., Wharrad, H., Taylor, M., & Ferguson, M. A. (2020). The development of an mHealth educational intervention for first-time hearing aid users: Combining theoretical and ecologically valid approaches. *International Journal of Audiology*, *59*(7), 492–500.

[41] Mann, D. M., Quintiliani, L. M., Reddy, S., Kitos, N. R., & Weng, M. (2014). Dietary approaches to stop hypertension: lessons learned from a case study on the development of an mHealth behavior change system. *JMIR MHealth and UHealth*, *2*(4), e41.

[42] Mathenjwa, T., Adeagbo, O., Zuma, T., Dikgale, K., Zeitlin, A., Matthews, P., Seeley, J., Wyke, S., Tanser, F., & Shahmanesh, M. (2020). Development and Acceptability of a Tablet-Based App to Support Men to Link to HIV Care: Mixed Methods Approach. *JMIR MHealth and UHealth*, *8*(11), e17549.

[43] McCarthy, O. L., Wazwaz, O., Calderon, V. O., Jado, I., Saibov, S., Stavridis, A., Gallardo, J. L., Tokhirov, R., Adada, S., & Huaynoca, S. (2018). Development of an intervention delivered by mobile phone aimed at decreasing unintended pregnancy among young people in three lower middle income countries. *BMC Public Health*, *18*(1), 1–15.

[44] Monteiro-Guerra, F., Signorelli, G. R., Tadas, S., Zubiete, E. D., Romero, O. R., Fernandez-Luque, L., & Caulfield, B. (2020). A personalized physical activity coaching app for breast cancer survivors: design process and early prototype testing. *JMIR MHealth and UHealth*, *8*(7), e17552.

[45] Morrison, D., Mair, F. S., Chaudhuri, R., McGee-Lennon, M., Thomas, M., Thomson, N. C., Yardley, L., & Wyke, S. (2015). Details of development of the resource for adults with asthma in the RAISIN (randomized trial of an asthma internet self-management intervention) study. *BMC Medical Informatics and Decision Making*, *15*(1), 1–16.

[46] Mueller, J., Davies, A., Jay, C., Harper, S., Blackhall, F., Summers, Y., Harle, A., & Todd, C. (2019). Developing and testing a web‐based intervention to encourage early help‐seeking in people with symptoms associated with lung cancer. *British Journal of Health Psychology*, *24*(1), 31–65.

[47] Mummah, S. A., King, A. C., Gardner, C. D., & Sutton, S. (2016). Iterative development of Vegethon: a theory-based mobile app intervention to increase vegetable consumption. *International Journal of Behavioral Nutrition and Physical Activity*, *13*(1), 1–12.

[48] Nelson, L. A., Mayberry, L. S., Wallston, K., Kripalani, S., Bergner, E. M., & Osborn, C. Y. (2016). Development and usability of REACH: a tailored theory-based text messaging intervention for disadvantaged adults with type 2 diabetes. *JMIR Human Factors*, *3*(2), e6029.

[49] Newby, K. V, Brown, K. E., Bayley, J., Kehal, I., Caley, M., Danahay, A., Hunt, J., & Critchley, G. (2017). Development of an intervention to increase sexual health service uptake by young people. *Health Promotion Practice*, *18*(3), 391–399.

[50] Partridge, S. R., Raeside, R., Latham, Z., Singleton, A. C., Hyun, K., Grunseit, A., Steinbeck, K., & Redfern, J. (2019). ‘Not to Be Harsh but Try Less to Relate to “the Teens” and You’ll Relate to Them More’: Co-Designing Obesity Prevention Text Messages with Adolescents. *International Journal of Environmental Research and Public Health*, *16*(24), 4887.

[51] Peleg, M., Michalowski, W., Wilk, S., Parimbelli, E., Bonaccio, S., O’Sullivan, D., Michalowski, M., Quaglini, S., & Carrier, M. (2018). Ideating mobile health behavioral support for compliance to therapy for patients with chronic disease: a case study of atrial fibrillation management. *Journal of Medical Systems*, *42*(11), 1–15.

[52] Potzel, A. L., Gar, C., Seissler, J., & Lechner, A. (2021). A Smartphone App (TRIANGLE) to Change Cardiometabolic Risk Behaviors in Women Following Gestational Diabetes Mellitus: Intervention Mapping Approach. *JMIR MHealth and UHealth*, *9*(5), e26163.

[53] Quintiliani, L. M., Foster, M., & Oshry, L. J. (2019). Preferences of mHealth app features for weight management among breast cancer survivors from underserved populations. *Psycho-Oncology*, *28*(10), 2101.

[54] Reidy, C., Foster, C., & Rogers, A. (2020). A facilitated web-based self-management tool for people with type 1 diabetes using an insulin pump: intervention development using the behavior change wheel and theoretical domains framework. *Journal of Medical Internet Research*, *22*(5), e13980.

[55] Ribaut, J., Leppla, L., Teynor, A., Valenta, S., Dobbels, F., Zullig, L. L., & De Geest, S. (2020). Theory-driven development of a medication adherence intervention delivered by eHealth and transplant team in allogeneic stem cell transplantation: the SMILe implementation science project. *BMC Health Services Research*, *20*(1), 1–22.

[56] Rodrigues, A. M., Sniehotta, F. F., Birch-Machin, M. A., Olivier, P., & Araújo-Soares, V. (2017). Systematic and iterative development of a smartphone app to promote sun-protection among holidaymakers: design of a prototype and results of usability and acceptability testing. *JMIR Research Protocols*, *6*(6), e112.

[57] Rohde, A., Duensing, A., Dawczynski, C., Godemann, J., Lorkowski, S., & Brombach, C. (2019). An app to improve eating habits of adolescents and young adults (challenge to go): systematic development of a theory-based and target group–adapted mobile app intervention. *JMIR MHealth and UHealth*, *7*(8), e11575.

[58] Ross, X. S., Gunn, K. M., Patterson, P., & Olver, I. (2019). Development of a smartphone program to support adherence to oral chemotherapy in people with cancer. *Patient Preference and Adherence*, *13*, 2207.

[59] Sakakibara, B. M., Lear, S. A., Barr, S. I., Benavente, O., Goldsmith, C. H., Silverberg, N. D., Yao, J., & Eng, J. J. (2017). Development of a chronic disease management program for stroke survivors using intervention mapping: the stroke coach. *Archives of Physical Medicine and Rehabilitation*, *98*(6), 1195–1202.

[60] Scheerman, J. F. M., Van Empelen, P., Van Loveren, C., & Van Meijel, B. (2018). A mobile app (WhiteTeeth) to promote good oral health behavior among Dutch adolescents with fixed orthodontic appliances: intervention mapping approach. *JMIR MHealth and UHealth*, *6*(8), e163.

[61] Shoneye, C. L., Mullan, B., Begley, A., Pollard, C. M., Jancey, J., & Kerr, D. A. (2020). Design and Development of a Digital Weight Management Intervention (ToDAy): Qualitative Study. *JMIR MHealth and UHealth*, *8*(9), e17919.

[62] Simons, D., De Bourdeaudhuij, I., Clarys, P., De Cocker, K., Vandelanotte, C., & Deforche, B. (2018). A smartphone app to promote an active lifestyle in lower-educated working young adults: development, usability, acceptability, and feasibility study. *JMIR MHealth and UHealth*, *6*(2), e8287.

[63] Sittig, S., Wang, J., Iyengar, S., Myneni, S., & Franklin, A. (2020). Incorporating behavioral trigger messages into a mobile health app for chronic disease management: randomized clinical feasibility trial in diabetes. *JMIR MHealth and UHealth*, *8*(3), e15927.

[64] Sporrel, K., De Boer, R. D. D., Wang, S., Nibbeling, N., Simons, M., Deutekom, M., Ettema, D., Castro, P. C., Dourado, V. Z., & Kröse, B. (2020). The Design and Development of a Personalized Leisure Time Physical Activity Application Based on Behavior Change Theories, End-User Perceptions, and Principles From Empirical Data Mining. *Frontiers in Public Health*, *8*.

[65] Stephenson, A., Garcia-Constantino, M., McDonough, S. M., Murphy, M. H., Nugent, C. D., & Mair, J. L. (2020). Iterative four-phase development of a theory-based digital behaviour change intervention to reduce occupational sedentary behaviour. *Digital Health*, *6*, 2055207620913410.

[66] Toefy, Y., Skinner, D., & Thomsen, S. (2016). “Please Don’t Send Us Spam!” A Participative, Theory-Based Methodology for Developing an mHealth Intervention. *JMIR MHealth and UHealth*, *4*(3), e6041.

[67] Tonkin, E., Jeffs, L., Wycherley, T. P., Maher, C., Smith, R., Hart, J., Cubillo, B., & Brimblecombe, J. (2017). A smartphone app to reduce sugar-sweetened beverage consumption among young adults in Australian remote indigenous communities: design, formative evaluation and user-testing. *JMIR MHealth and UHealth*, *5*(12), e8651.

[68] van Agteren, J. E. M., Lawn, S., Bonevski, B., & Smith, B. J. (2018). Kick. it: the development of an evidence-based smoking cessation smartphone app. *Translational Behavioral Medicine*, *8*(2), 243–267.

[69] Verbiest, M. E. A., Corrigan, C., Dalhousie, S., Firestone, R., Funaki, T., Goodwin, D., Grey, J., Henry, A., Humphrey, G., & Jull, A. (2019). Using codesign to develop a culturally tailored, behavior change mHealth intervention for indigenous and other priority communities: A case study in New Zealand. *Translational Behavioral Medicine*, *9*(4), 720–736.

[70] Walsh, D. M. J., Moran, K., Cornelissen, V., Buys, R., Claes, J., Zampognaro, P., Melillo, F., Maglaveras, N., Chouvarda, I., & Triantafyllidis, A. (2019). The development and codesign of the PATHway intervention: a theory-driven eHealth platform for the self-management of cardiovascular disease. *Translational Behavioral Medicine*, *9*(1), 76–98.

[71] Warren, I., Meads, A., Whittaker, R., Dobson, R., & Ameratunga, S. (2018). Behavior change for youth drivers: design and development of a smartphone-based app (BackPocketDriver). *JMIR Formative Research*, *2*(2), e9660.

[72] Waterlander, W., Whittaker, R., McRobbie, H., Dorey, E., Ball, K., Maddison, R., Smith, K. M., Crawford, D., Jiang, Y., & Gu, Y. (2014). Development of an evidence-based mHealth weight management program using a formative research process. *JMIR MHealth and UHealth*, *2*(3), e2850.

[73] Wen, K.-Y., Miller, S. M., Kilby, L., Fleisher, L., Belton, T. D., Roy, G., & Hernandez, E. (2014). Preventing postpartum smoking relapse among inner city women: development of a theory-based and evidence-guided text messaging intervention. *JMIR Research Protocols*, *3*(2), e3059.

[74] White, B. K., Martin, A., White, J. A., Burns, S. K., Maycock, B. R., Giglia, R. C., & Scott, J. A. (2016). Theory-based design and development of a socially connected, gamified mobile app for men about breastfeeding (Milk Man). *JMIR MHealth and UHealth*, *4*(2), e5652.

[75] Zuidema, R. M., van Gaal, B. G. I., van Dulmen, S., Repping-Wuts, H., & Schoonhoven, L. (2015). An online tailored self-management program for patients with rheumatoid arthritis: a developmental study. *JMIR Research Protocols*, *4*(4), e140.
