# Supplementary material for: Integrating Behavioral Science and Design Thinking to Develop Mobile Health Interventions: Systematic Scoping Review
Source: JMIR Mhealth Uhealth. 2022 Mar 16;10(3):e35799. doi: 10.2196/35799 (PMC8968622; doi:10.2196/35799)
Supplement: Multimedia Appendix 5 [file mhealth_v10i3e35799_app5.doc]

**Multimedia Appendix 5: Theories, Models and Frameworks used in Design***

| **Guide (“Procedural”)**  Theories, Models and Frameworks studies used to guide the design process in a stepwise manner. | | |
| --- | --- | --- |
| **Theories, Models and Frameworks** | **References** | **n** |
| Intervention Mapping | [1][49][46][63][32][20][48][21][42] [52] [66][50][56][53][29] [62] [25] [55] | 18 |
| Behavior Change Wheel | [1][2][4] [66][5] [68][7][8] [9] [10] [12] [13] [14] [15] [28] | 15 |
| User-Centred Design | [18] [54][40][6][22] [68] [28] [7][37][61] [8] [11] [15] [24] [74] | 15 |
| MRC Framework for Complex Interventions | [64][59] [4][68][72] [15] [39] | 7 |
| IDEAS Framework | [44][75] [45][9] [13] [74] | 6 |
| Behavioral Intervention Technology Framework | [3][65] [9] [10] [55] | 5 |
| Person-Based Approach | [3][35][47] [10] [12] | 5 |
| Agile Development | [64] [12] [14] | 3 |
| Co-Design Framework | [34] [30] [69] | 3 |
| Development and Evaluation Framework for mHealth Interventions | [65] [28] [46] | 3 |
| ORBIT Model (Obesity-Related Behavioral Intervention Trials) | [70] | 1 |
| Computer Tailoring Method | [50] | 1 |
| Human-Centred Design | [39] | 1 |
| Research Through Design | [58] | 1 |
| Shah’s Methodological Framework | [18] | 1 |
| Community Embedded Iterative Design Framework | [19] | 1 |
| Causal Modelling Behavior Change Conceptual Framework | [21] | 1 |
| Double Diamond Framework | [67] | 1 |
| Center for eHealth Research and Disease Management (CeHRes) Roadmap | [67] | 1 |
| Collaborative Product Development Process | [7] | 1 |
| The Action Research Approach | [38] | 1 |

| **Conceptualize (“Diagnostic”)**  Theories, Models and Frameworks studies used to conceptualize the behavior change problem (i.e., behavior change barriers and facilitators) the mHealth DBCI should address. | | |
| --- | --- | --- |
| **Theories, Models and Frameworks** | **References** | **n** |
| Capability-Opportunity-Motivation-Behavior (COM-B) Model | [1] [2] [3] [4] [5] [6] [7] [8][9][10] [11] [12] [13] [14] [15] | 15 |
| Social Cognitive Theory | [16][17] [18][19] [20] [21] [22][23] [24] [25] [26] [27] [28] | 13 |
| Theoretical Domains Framework | [1] [4] [29] [8] [10] [11] [12] [14] [15] [30] | 10 |
| Theory of Planned Behavior | [31] [32][33][34] [35][36] [37][38] [39] | 9 |
| Transtheoretical Model | [27] [40][41] [42][43] [44] [45] [25] [20] | 9 |
| Self-Determination Theory | [18] [46] [47] [48] [44] [23] [37] | 7 |
| Health Action Process Approach | [49] [2] [27] [20] [50] [38] | 6 |
| Self-Regulation Theory | [49] [19] [20] [25] [27] | 5 |
| Information-Motivation-Behavioral Skills Model | [51] [52] [53] [24] | 4 |
| Integrated Behavior Change Model | [52] [50] [55] | 3 |
| Elaboration Likelihood Model | [54] [6] | 2 |
| Fogg Behavioral Model | [44] [22] | 2 |
| Attitude-Social Influence-Self Efficacy Model | [46] [56] | 2 |
| Health Belief Model | [57] [37] | 2 |
| Patient Engagement Framework | [60] [53] | 2 |
| Narrative Transportation Theory | [23] | 1 |
| Habit - Identity Loop | [58] | 1 |
| Personalization Theory | [18] | 1 |
| Dual-Process Approach | [33] | 1 |
| Prototype Willingness Model | [33] | 1 |
| Normalization Process Theory | [59] | 1 |
| Control Theory | [21] | 1 |
| Stress-Vulnerability Model | [42] | 1 |
| Self-Regulatory Action Planning and Coping Model | [36] | 1 |
| Contemplation-Action-Maintenance Model | [50] | 1 |
| Goal Setting Theory | [37] | 1 |
| Cognitive-Social Health Information Processing Model | [61] | 1 |
| Integrated Change Model | [62] | 1 |
| Appreciative Inquiry | [25] | 1 |
| Unified Theory of Acceptance and Use of Technology | [28] | 1 |

| **Identify (“Prescriptive”)**  Theories, Models and Frameworks studies used to identify relevant behavior change techniques and strategies for the mHealth DBCI. | | |
| --- | --- | --- |
| **Theories, Models and Frameworks** | **References** | **n** |
| Behavior Change Technique (BCT) Taxonomy | [1] [49] [18] [46] [63][54] [2] [32] [33] [19] [64] [3] [40] [59] [35] [47] [21] [4] [65][66] [5] [50] [6] [44][67] [68] [29] [28] [7] [60][69] [70][71] [62] [39] [72] [8] [10] [12] [13] [14] [73] [15] [30] [55] [24] [42] [4] [74] | 49 |
| Gamification Principles | [2] [33] [64] [75] [23] [37] [70] [13] [58] [74] | 10 |
| Behavioral Intervention Technology Framework | [3][65] [9] [10] [55] | 5 |
| Persuasive System Design | [67] [22] [29] [27] | 4 |
| CALO-RE Taxonomy | [18] | 1 |
| Human-Computer Interaction Techniques | [47] | 1 |
| Motivational Interviewing Approach | [48] | 1 |
| Enhanced Active Choice | [57] | 1 |
| Social Nudging Theories | [57] | 1 |
| Model of Actional Feedback | [57] | 1 |
| Behavioral Motivational Interviewing | [45] | 1 |
| Ecological Momentary Interventions | [45] | 1 |

| **Evaluate (“Appraisal”)**  Theories, Models and Frameworks studies used to evaluate design ideas and solutions for their feasibility, applicability, and potential effectiveness. | | |
| --- | --- | --- |
| **Theories, Models and Frameworks** | **References** | **n** |
| System Usability Scale (SUS) Tool | [31] [63] [54] [40] [5] [60] [71] [5] [74] | 9 |
| BCT Taxonomy | [40] [59] [21] [12] [73] [66] [54] | 7 |
| Mobile App Rating Scale (MARS) Tool | [18] [63] [7] [26] [24] [7] | 6 |
| APEASE Criteria | [1] [2] [4] [68] [14] | 5 |
| PRECEDE-PROCEED Model | [42] [56] [29] [62] | 4 |
| Nielsen’s Usability Heuristics Checklist | [68] [60] [5] [37] | 4 |
| Self-Report Habit Index | [58] | 1 |
| App Behavior Change Scale (ABACUS) Tool | [18] | 1 |
| MoSCoW Methods | [34] | 1 |
| Transtheoretical Model | [40] | 1 |
| Self-Determination Theory | [47] | 1 |
| Moville’s Honeycomb Model of User Experience | [5] | 1 |
| COM-B Model | [5] | 1 |
| TAM | [44] | 1 |
| Unified Theory of Acceptance and Use of Technology | [28] | 1 |
| 5-E Approach | [10] | 1 |
| Social Cognitive Theory | [16] | 1 |

* The lists above are not meant to be exhaustive of all the theories, models and frameworks studies drew from. The theories, models and frameworks cited above are representative of what was explicitly mentioned and clearly described by studies. Studies often elected to draw on broad literature reviews, group brainstorming, practice guidelines, user feedback and other methods to conceptualize, identify, evaluate, and guide their work. The categories presented here (conceptualize, identify, evaluate and guide) are also not meant to be mutually exclusive. While overlap exists between these classifications, conceptualizing the differences between certain types of theories, models and frameworks can help facilitate selection of tools to guide mHealth DBCI design. More information on the approaches and methods studies used throughout mHealth DBCI design can be seen in Appendix 4).

**References**

[1] C. Reidy, C. Foster, and A. Rogers, “A facilitated web-based self-management tool for people with type 1 diabetes using an insulin pump: intervention development using the behavior change wheel and theoretical domains framework,” *J. Med. Internet Res.*, vol. 22, no. 5, p. e13980, 2020.

[2] A. Rohde, A. Duensing, C. Dawczynski, J. Godemann, S. Lorkowski, and C. Brombach, “An app to improve eating habits of adolescents and young adults (challenge to go): systematic development of a theory-based and target group–adapted mobile app intervention,” *JMIR mHealth uHealth*, vol. 7, no. 8, p. e11575, 2019.

[3] C. L. Shoneye, B. Mullan, A. Begley, C. M. Pollard, J. Jancey, and D. A. Kerr, “Design and Development of a Digital Weight Management Intervention (ToDAy): Qualitative Study,” *JMIR mHealth uHealth*, vol. 8, no. 9, p. e17919, 2020.

[4] I. B. Félix *et al.*, “Development of a complex intervention to improve adherence to antidiabetic medication in older people using an anthropomorphic virtual assistant software,” *Front. Pharmacol.*, vol. 10, p. 680, 2019.

[5] W.-J. Chang, S.-Y. Lo, C.-L. Kuo, Y.-L. Wang, and H.-C. Hsiao, “Development of an intervention tool for precision oral self-care: Personalized and evidence-based practice for patients with periodontal disease,” *PLoS One*, vol. 14, no. 11, p. e0225453, 2019.

[6] D. M. Mann, L. M. Quintiliani, S. Reddy, N. R. Kitos, and M. Weng, “Dietary approaches to stop hypertension: lessons learned from a case study on the development of an mHealth behavior change system,” *JMIR mHealth uHealth*, vol. 2, no. 4, p. e41, 2014.

[7] J. Chen, E. Ho, Y. Jiang, R. Whittaker, T. Yang, and C. Bullen, “Mobile Social Network–Based Smoking Cessation Intervention for Chinese Male Smokers: Pilot Randomized Controlled Trial,” *JMIR mHealth uHealth*, vol. 8, no. 10, p. e17522, 2020.

[8] K. E. Curtis, S. Lahiri, and K. E. Brown, “Targeting parents for childhood weight management: development of a theory-driven and user-centered healthy eating app,” *JMIR mHealth uHealth*, vol. 3, no. 2, p. e3857, 2015.

[9] K. Sporrel *et al.*, “The Design and Development of a Personalized Leisure Time Physical Activity Application Based on Behavior Change Theories, End-User Perceptions, and Principles From Empirical Data Mining,” *Front. Public Heal.*, vol. 8, 2020.

[10] D. M. J. Walsh *et al.*, “The development and codesign of the PATHway intervention: a theory-driven eHealth platform for the self-management of cardiovascular disease,” *Transl. Behav. Med.*, vol. 9, no. 1, pp. 76–98, 2019.

[11] D. W. Maidment, N. S. Coulson, H. Wharrad, M. Taylor, and M. A. Ferguson, “The development of an mHealth educational intervention for first-time hearing aid users: Combining theoretical and ecologically valid approaches,” *Int. J. Audiol.*, vol. 59, no. 7, pp. 492–500, 2020.

[12] C. Garnett, D. Crane, R. West, J. Brown, and S. Michie, “The development of Drink Less: an alcohol reduction smartphone app for excessive drinkers,” *Transl. Behav. Med.*, vol. 9, no. 2, pp. 296–307, 2019.

[13] G. A. Hendrie, G. James-Martin, G. Williams, E. Brindal, B. Whyte, and A. Crook, “The development of VegEze: smartphone app to increase vegetable consumption in Australian adults,” *JMIR Form. Res.*, vol. 3, no. 1, p. e10731, 2019.

[14] J. Ribaut *et al.*, “Theory-driven development of a medication adherence intervention delivered by eHealth and transplant team in allogeneic stem cell transplantation: the SMILe implementation science project,” *BMC Health Serv. Res.*, vol. 20, no. 1, pp. 1–22, 2020.

[15] Y. J. G. Korpershoek, S. Hermsen, L. Schoonhoven, M. J. Schuurmans, and J. C. A. Trappenburg, “User-centered design of a mobile health intervention to enhance exacerbation-related self-management in patients with chronic obstructive pulmonary disease (copilot): mixed methods study,” *J. Med. Internet Res.*, vol. 22, no. 6, p. e15449, 2020.

[16] R. P. Joseph *et al.*, “A culturally relevant smartphone-delivered physical activity intervention for African American women: Development and initial usability tests of smart walk,” *JMIR mHealth uHealth*, vol. 8, no. 3, p. e15346, 2020.

[17] S. Hales *et al.*, “A mixed-methods approach to the development, refinement, and pilot testing of social networks for improving healthy behaviors,” *JMIR Hum. factors*, vol. 3, no. 1, p. e4512, 2016.

[18] F. Monteiro-Guerra *et al.*, “A personalized physical activity coaching app for breast cancer survivors: design process and early prototype testing,” *JMIR mHealth uHealth*, vol. 8, no. 7, p. e17552, 2020.

[19] M. P. Buman *et al.*, “BeWell24: development and process evaluation of a smartphone ‘app’ to improve sleep, sedentary, and active behaviors in US Veterans with increased metabolic risk,” *Transl. Behav. Med.*, vol. 6, no. 3, pp. 438–448, 2016.

[20] H. Brendryen, A. B. Johansen, S. Nesvåg, G. Kok, and F. Duckert, “Constructing a theory-and evidence-based treatment rationale for complex eHealth interventions: Development of an online alcohol intervention using an intervention mapping approach,” *JMIR Res. Protoc.*, vol. 2, no. 1, p. e6, 2013.

[21] B. M. Sakakibara *et al.*, “Development of a chronic disease management program for stroke survivors using intervention mapping: the stroke coach,” *Arch. Phys. Med. Rehabil.*, vol. 98, no. 6, pp. 1195–1202, 2017.

[22] S. Sittig, J. Wang, S. Iyengar, S. Myneni, and A. Franklin, “Incorporating behavioral trigger messages into a mobile health app for chronic disease management: randomized clinical feasibility trial in diabetes,” *JMIR mHealth uHealth*, vol. 8, no. 3, p. e15927, 2020.

[23] B. F. Fuemmeler *et al.*, “Mila blooms: a mobile phone application and behavioral intervention for promoting physical activity and a healthy diet among adolescent survivors of childhood cancer,” *Games Health J.*, vol. 9, no. 4, pp. 279–289, 2020.

[24] M. D. Adu, U. H. Malabu, A. E. O. Malau-Aduli, and B. S. Malau-Aduli, “The development of My Care Hub mobile-phone app to support self-management in Australians with type 1 or type 2 diabetes,” *Sci. Rep.*, vol. 10, no. 1, pp. 1–10, 2020.

[25] P. Dalum, C. L. Brandt, L. Skov-Ettrup, J. Tolstrup, and G. Kok, “The systematic development of an internet-based smoking cessation intervention for adults,” *Health Promot. Pract.*, vol. 17, no. 4, pp. 490–500, 2016.

[26] B. K. White *et al.*, “Theory-based design and development of a socially connected, gamified mobile app for men about breastfeeding (Milk Man),” *JMIR mHealth uHealth*, vol. 4, no. 2, p. e5652, 2016.

[27] K. Antypas and S. C. Wangberg, “Combining users’ needs with health behavior models in designing an internet-and mobile-based intervention for physical activity in cardiac rehabilitation,” *JMIR Res. Protoc.*, vol. 3, no. 1, p. e4, 2014.

[28] O. Duff *et al.*, “MedFit app, a behavior-changing, theoretically informed mobile app for patient self-management of cardiovascular disease: user-centered development,” *JMIR Form. Res.*, vol. 2, no. 1, p. e9550, 2018.

[29] J. E. M. van Agteren, S. Lawn, B. Bonevski, and B. J. Smith, “Kick. it: the development of an evidence-based smoking cessation smartphone app,” *Transl. Behav. Med.*, vol. 8, no. 2, pp. 243–267, 2018.

[30] M. E. A. Verbiest *et al.*, “Using codesign to develop a culturally tailored, behavior change mHealth intervention for indigenous and other priority communities: A case study in New Zealand,” *Transl. Behav. Med.*, vol. 9, no. 4, pp. 720–736, 2019.

[31] E. Tonkin *et al.*, “A smartphone app to reduce sugar-sweetened beverage consumption among young adults in Australian remote indigenous communities: design, formative evaluation and user-testing,” *JMIR mHealth uHealth*, vol. 5, no. 12, p. e8651, 2017.

[32] R. M. Zuidema, B. G. I. van Gaal, S. van Dulmen, H. Repping-Wuts, and L. Schoonhoven, “An online tailored self-management program for patients with rheumatoid arthritis: a developmental study,” *JMIR Res. Protoc.*, vol. 4, no. 4, p. e140, 2015.

[33] I. Warren, A. Meads, R. Whittaker, R. Dobson, and S. Ameratunga, “Behavior change for youth drivers: design and development of a smartphone-based app (BackPocketDriver),” *JMIR Form. Res.*, vol. 2, no. 2, p. e9660, 2018.

[34] R. Lipson-Smith *et al.*, “Co-design of a consultation audio-recording mobile app for people with cancer: the SecondEars app,” *JMIR Form. Res.*, vol. 3, no. 1, p. e11111, 2019.

[35] J. Mueller *et al.*, “Developing and testing a web‐based intervention to encourage early help‐seeking in people with symptoms associated with lung cancer,” *Br. J. Health Psychol.*, vol. 24, no. 1, pp. 31–65, 2019.

[36] W. Waterlander *et al.*, “Development of an evidence-based mHealth weight management program using a formative research process,” *JMIR mHealth uHealth*, vol. 2, no. 3, p. e2850, 2014.

[37] G. Giunti, V. Mylonopoulou, and O. R. Romero, “More stamina, a gamified mhealth solution for persons with multiple sclerosis: research through design,” *JMIR mHealth uHealth*, vol. 6, no. 3, p. e9437, 2018.

[38] Y. Toefy, D. Skinner, and S. Thomsen, “‘Please Don’t Send Us Spam!’ A Participative, Theory-Based Methodology for Developing an mHealth Intervention,” *JMIR mHealth uHealth*, vol. 4, no. 3, p. e6041, 2016.

[39] H. Hawley-Hague *et al.*, “Smartphone apps to support falls rehabilitation exercise: app development and usability and acceptability study,” *JMIR mHealth uHealth*, vol. 8, no. 9, p. e15460, 2020.

[40] S. Gabrielli *et al.*, “Design of a mobile app for nutrition education (TreC-LifeStyle) and formative evaluation with families of overweight children,” *JMIR mHealth uHealth*, vol. 5, no. 4, p. e7080, 2017.

[41] M. K. Lee, H.-A. Park, Y. H. Yun, and Y. J. Chang, “Development and formative evaluation of a web-based self-management exercise and diet intervention program with tailored motivation and action planning for cancer survivors,” *JMIR Res. Protoc.*, vol. 2, no. 1, p. e2331, 2013.

[42] T. A. A. Beentjes, B. G. I. van Gaal, P. J. J. Goossens, and L. Schoonhoven, “Development of an e-supported illness management and recovery programme for consumers with severe mental illness using intervention mapping, and design of an early cluster randomized controlled trial,” *BMC Health Serv. Res.*, vol. 16, no. 1, pp. 1–9, 2015.

[43] L. Hebden, A. Cook, H. P. Van Der Ploeg, and M. Allman-Farinelli, “Development of smartphone applications for nutrition and physical activity behavior change,” *JMIR Res. Protoc.*, vol. 1, no. 2, p. e2205, 2012.

[44] M. Peleg *et al.*, “Ideating mobile health behavioral support for compliance to therapy for patients with chronic disease: a case study of atrial fibrillation management,” *J. Med. Syst.*, vol. 42, no. 11, pp. 1–15, 2018.

[45] D. M. Kazemi, B. Borsari, M. J. Levine, K. A. Lamberson, and B. Dooley, “REMIT: Development of a mHealth theory-based intervention to decrease heavy episodic drinking among college students.,” *Addict. Res. Theory*, vol. 26, no. 5, pp. 377–385, 2018.

[46] D. Simons, I. De Bourdeaudhuij, P. Clarys, K. De Cocker, C. Vandelanotte, and B. Deforche, “A smartphone app to promote an active lifestyle in lower-educated working young adults: development, usability, acceptability, and feasibility study,” *JMIR mHealth uHealth*, vol. 6, no. 2, p. e8287, 2018.

[47] T. Mathenjwa *et al.*, “Development and Acceptability of a Tablet-Based App to Support Men to Link to HIV Care: Mixed Methods Approach,” *JMIR mHealth uHealth*, vol. 8, no. 11, p. e17549, 2020.

[48] J. M. J. Coumans, C. A. W. Bolman, S. A. H. Friederichs, A. Oenema, and L. Lechner, “Development and testing of a personalized web-based diet and physical activity intervention based on motivational interviewing and the self-determination theory: protocol for the mylifestylecoach randomized controlled trial,” *JMIR Res. Protoc.*, vol. 9, no. 2, p. e14491, 2020.

[49] J. F. M. Scheerman, P. Van Empelen, C. Van Loveren, and B. Van Meijel, “A mobile app (WhiteTeeth) to promote good oral health behavior among Dutch adolescents with fixed orthodontic appliances: intervention mapping approach,” *JMIR mHealth uHealth*, vol. 6, no. 8, p. e163, 2018.

[50] Y. Chen *et al.*, “Development of interventions for an intelligent and individualized mobile health care system to promote healthy diet and physical activity: using an intervention mapping framework,” *BMC Public Health*, vol. 19, no. 1, pp. 1–16, 2019.

[51] L. A. Nelson, L. S. Mayberry, K. Wallston, S. Kripalani, E. M. Bergner, and C. Y. Osborn, “Development and usability of REACH: a tailored theory-based text messaging intervention for disadvantaged adults with type 2 diabetes,” *JMIR Hum. Factors*, vol. 3, no. 2, p. e6029, 2016.

[52] O. L. McCarthy *et al.*, “Development of an intervention delivered by mobile phone aimed at decreasing unintended pregnancy among young people in three lower middle income countries,” *BMC Public Health*, vol. 18, no. 1, pp. 1–15, 2018.

[53] P. Athilingam, J. M. Clochesy, and M. A. Labrador, “Intervention mapping approach in the design of an interactive mobile health application to improve self-care in heart failure,” *CIN Comput. Informatics, Nurs.*, vol. 36, no. 2, pp. 90–97, 2018.

[54] A. DeSmet, I. De Bourdeaudhuij, S. Chastin, G. Crombez, R. Maddison, and G. Cardon, “Adults’ preferences for behavior change techniques and engagement features in a mobile app to promote 24-hour movement behaviors: Cross-sectional survey study,” *JMIR mHealth uHealth*, vol. 7, no. 12, p. e15707, 2019.

[55] A. Direito *et al.*, “Using the intervention mapping and behavioral intervention technology frameworks: development of an mHealth intervention for physical activity and sedentary behavior change,” *Heal. Educ. Behav.*, vol. 45, no. 3, pp. 331–348, 2018.

[56] C. M. den Bakker *et al.*, “Electronic health program to empower patients in returning to normal activities after general surgical and gynecological procedures: Intervention mapping as a useful method for further development,” *J. Med. Internet Res.*, vol. 21, no. 2, p. e9938, 2019.

[57] B. Bogale *et al.*, “Development of a targeted client communication intervention to women using an electronic maternal and child health registry: a qualitative study,” *BMC Med. Inform. Decis. Mak.*, vol. 20, no. 1, pp. 1–12, 2020.

[58] F. Hooglugt and G. D. S. Ludden, “A mobile app adopting an identity focus to promote physical activity (MoveDaily): iterative design study,” *JMIR mHealth uHealth*, vol. 8, no. 6, p. e16720, 2020.

[59] D. Morrison *et al.*, “Details of development of the resource for adults with asthma in the RAISIN (randomized trial of an asthma internet self-management intervention) study,” *BMC Med. Inform. Decis. Mak.*, vol. 15, no. 1, pp. 1–16, 2015.

[60] L. Carter, D. Rogith, A. Franklin, and S. Myneni, “NewCope: A Theory-Linked Mobile Application for Stress Education and Management,” *Stud. Health Technol. Inform.*, vol. 264, p. 1150, 2019.

[61] K.-Y. Wen *et al.*, “Preventing postpartum smoking relapse among inner city women: development of a theory-based and evidence-guided text messaging intervention,” *JMIR Res. Protoc.*, vol. 3, no. 2, p. e3059, 2014.

[62] M. M. Engelen *et al.*, “Evaluation of a Web-Based Self-Management Program for Patients With Cardiovascular Disease: Explorative Randomized Controlled Trial.,” *J. Med. Internet Res.*, vol. 22, no. 7, p. e17422, 2020.

[63] A. L. Potzel, C. Gar, J. Seissler, and A. Lechner, “A Smartphone App (TRIANGLE) to Change Cardiometabolic Risk Behaviors in Women Following Gestational Diabetes Mellitus: Intervention Mapping Approach,” *JMIR mHealth uHealth*, vol. 9, no. 5, p. e26163, 2021.

[64] E. A. Edwards *et al.*, “Creating a theoretically grounded, gamified health app: Lessons from developing the Cigbreak smoking cessation mobile phone game,” *JMIR serious games*, vol. 6, no. 4, p. e10252, 2018.

[65] X. S. Ross, K. M. Gunn, P. Patterson, and I. Olver, “Development of a smartphone program to support adherence to oral chemotherapy in people with cancer,” *Patient Prefer. Adherence*, vol. 13, p. 2207, 2019.

[66] K. V Newby *et al.*, “Development of an intervention to increase sexual health service uptake by young people,” *Health Promot. Pract.*, vol. 18, no. 3, pp. 391–399, 2017.

[67] R. A. Asbjørnsen *et al.*, “Identifying persuasive design principles and behavior change techniques supporting end user values and needs in ehealth interventions for long-term weight loss maintenance: qualitative study,” *J. Med. Internet Res.*, vol. 22, no. 11, p. e22598, 2020.

[68] A. Stephenson, M. Garcia-Constantino, S. M. McDonough, M. H. Murphy, C. D. Nugent, and J. L. Mair, “Iterative four-phase development of a theory-based digital behavior change intervention to reduce occupational sedentary behavior,” *Digit. Heal.*, vol. 6, p. 2055207620913410, 2020.

[69] S. R. Partridge *et al.*, “‘Not to Be Harsh but Try Less to Relate to “the Teens” and You’ll Relate to Them More’: Co-Designing Obesity Prevention Text Messages with Adolescents,” *Int. J. Environ. Res. Public Health*, vol. 16, no. 24, p. 4887, 2019.

[70] J. M. Brown *et al.*, “Optimizing child nutrition education with the Foodbot Factory mobile health app: formative evaluation and analysis,” *JMIR Form. Res.*, vol. 4, no. 4, p. e15534, 2020.

[71] L. M. Quintiliani, M. Foster, and L. J. Oshry, “Preferences of mHealth app features for weight management among breast cancer survivors from underserved populations,” *Psychooncology.*, vol. 28, no. 10, p. 2101, 2019.

[72] A. M. Rodrigues, F. F. Sniehotta, M. A. Birch-Machin, P. Olivier, and V. Araújo-Soares, “Systematic and iterative development of a smartphone app to promote sun-protection among holidaymakers: design of a prototype and results of usability and acceptability testing,” *JMIR Res. Protoc.*, vol. 6, no. 6, p. e112, 2017.

[73] Y. K. Bartlett, A. Farmer, R. Rea, and D. P. French, “Use of brief messages based on behavior change techniques to encourage medication adherence in people with type 2 diabetes: developmental studies,” *J. Med. Internet Res.*, vol. 22, no. 5, p. e15989, 2020.

[74] B. Kim *et al.*, “PuzzleWalk: A theory-driven iterative design inquiry of a mobile game for promoting physical activity in adults with autism spectrum disorder,” *PLoS One*, vol. 15, no. 9, p. e0237966, 2020.

[75] S. A. Mummah, A. C. King, C. D. Gardner, and S. Sutton, “Iterative development of Vegethon: a theory-based mobile app intervention to increase vegetable consumption,” *Int. J. Behav. Nutr. Phys. Act.*, vol. 13, no. 1, pp. 1–12, 2016.
