# Supplementary material for: Integrating Behavioral Science and Design Thinking to Develop Mobile Health Interventions: Systematic Scoping Review
Source: JMIR Mhealth Uhealth. 2022 Mar 16;10(3):e35799. doi: 10.2196/35799 (PMC8968622; doi:10.2196/35799)
Supplement: Multimedia Appendix 6 [file mhealth_v10i3e35799_app6.pdf]

Behavioral Design Thinking

Integrating methods from Behavioral Design (BD) and Design Thinking (DT) to develop mHealth interventions that more effectively engage users

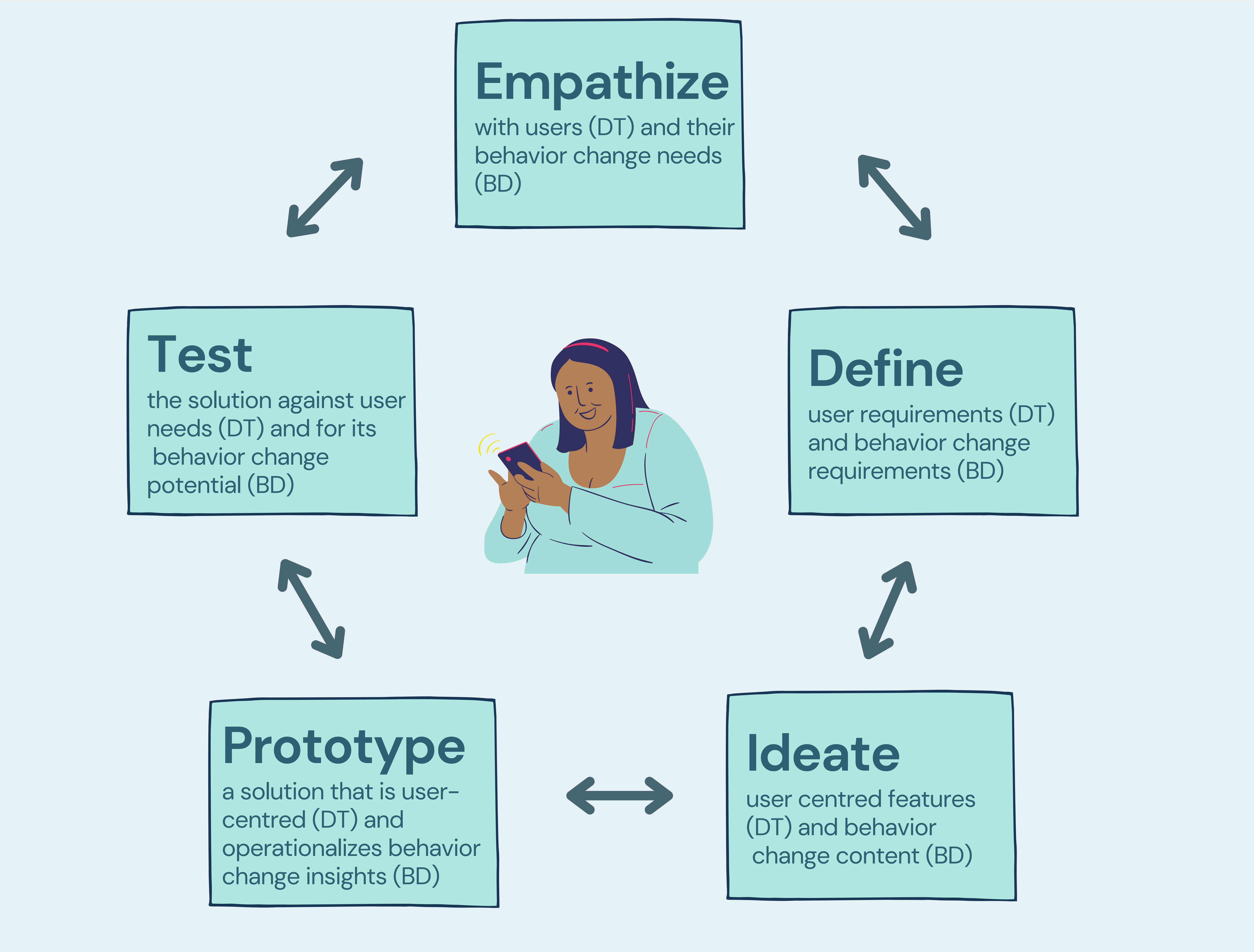

The Behavioral Design Thinking Approach is based on a review of 75 studies that combined insights from Behavioral Design (BD) and Design Thinking (DT) throughout their mHealth design process [1-75]. The approaches and methods studies used to integrate BD and DT at each stage of the design process are described, along with examples.*

**The five stages outlined below are not meant to be mutually exclusive or followed in a linear fashion. The five stages have been used to organize and make sense of the vast range of methods and approaches available to design teams. The methods and approaches listed are also not meant to be exhaustive of what can be utilized during Behavioral Design Thinking.*

Empathize

with users and their behavior change needs

Summary of approaches and methods from 75 studies [1-75]

Methods
to empathize

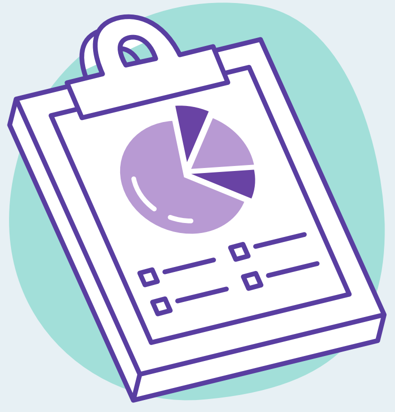

- **Primary Research:** Interviews, focus groups, surveys, workshops
- **Secondary Research:** Literature reviews, secondary data analyses, reviews of mobile applications, reviews of best-practice guidelines

Who
to empathize with

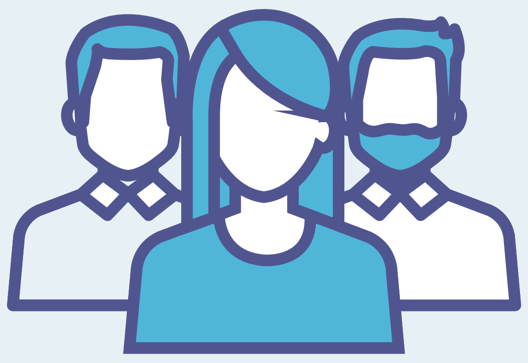

- **End users:** Patients and/or the public
- **Key stakeholders:** Healthcare workers, community partners, behavioral scientists, design scientists, technology developers

Key Concepts when "Empathizing"

DT

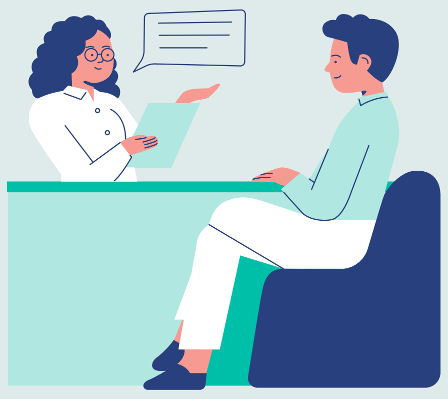

Understand the users
who will perform the behavior

↔

Understand the behavior
that the users will perform

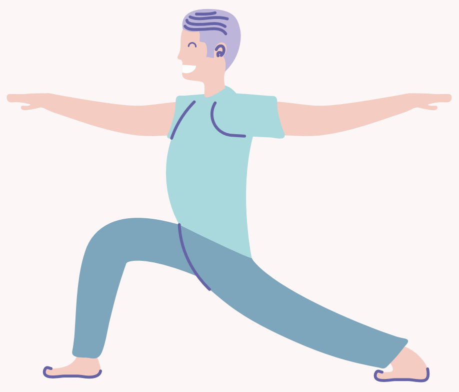

BD

- **What are users experiences with** the health issue, health behavior changes, health interventions, mobile applications?
- **What are users' perceptions and beliefs about** the health issue, health behavior changes, health interventions, mobile applications?
- **What are users needs and preferences regarding** future intervention content, intervention features, behaviors to perform, and supports to receive ?

- **What health behavior changes** are most appropriate to target?
- **Which behavioral theories, models, and frameworks** will help conceptualize the behavior change issue?
- **What are the behavioral determinants** (barriers and facilitators) of the target behavior change?
- **What behavior change techniques and strategies** can help address the behavioral determinate?

Understand the context
that the users perform the behavior in

- **What are the current** behavioral norms, healthcare practices, mobile app trends, and healthcare interventions?
- **What factors (personal, social, environmental, structural)** may impact the health issue, behavior change adherence, and/or mobile application usage?
- **What aims and measures (clinical, usage, and behavioral)** are appropriate given the user context?

Empathize

with users and their behavior change needs

Examples from included studies [44,67,16]

Example 1:
A personalized physical activity coaching app for breast cancer survivors: design process and early prototype testing by Monteiro-Guerra et al., 2020 [44]

Stage 1 of Monteiro-Guerra et al's design process involved "User and Context Research". During this stage, the needs and requirements of breast cancer survivors were empathized with, especially regarding **content**, **expected benefits, features,** and **personalization** and **motivational aspects of the proposed physical activity (PA) tool.** To empathize with users, Monteiro-Guerra et al. performed **(1) a qualitative study with the target users** and **(2) a review of related literature.** The qualitative study involved a combination of **open-ended questions on PA adherence and technology interest** and **a slideshow presentation with examples of PA app features to obtain participants' thoughts and opinions** on the featured content. A rapid literature review was performed to complement the findings from the user study on **(1) barriers and facilitators for PA; (2) attitudes, needs, and preferences from breast cancer survivors on PA apps;** and **(3) information on behavioral theory used in PA interventions for breast cancer.**

Example 2:
A Smartphone App to Reduce Sugar-Sweetened Beverage Consumption Among Young Adults in Australian Remote Indigenous Communities: Design, Formative Evaluation and User-Testing by Tonkin et al., 2017 [67]

Stage 1 of Tonkin et al's design process involved "Formative Research". During this stage, Tonkin et al. aimed to **understand potential app features, content, and structure using:** (1) a simulated grocery-selection activity with think aloud (a "think aloud" shop); (2) a semistructured interview regarding eating behaviors; and (3) a questionnaire outlining current smartphone and app use and paper prototyping activity.
The "think aloud" shop activity was included to collect data related to the determinants of food choice for young adults living in remote Indigenous communities. These data were collected to identify key nutrition features and content for the app. **The semistructured interviews** aimed to collect data related to eating behavior and food attitudes in order to determine approaches to app design to best support behavior change. **The interview schedule was designed using the constructs of the Theory of Planned Behavior (TPB).** The **Smartphone Use Questionnaire** examined current smartphone and app use to inform foundational app functionality. **The paper prototyping method** directly involved target users in the concpetualization of app features and styling, using trigger materials to encourage participants to build an app prototype.

Example 3:
Targeting Parents for Childhood Weight Management: Development of a Theory-Driven and User-Centered Healthy Eating App by Curtis et al., 2015 [16]

Stage 1 of Curtis et al's design process involved "Understanding the Problem and User Preferences". **First, Curtis et al. defined the program in behavioral terms while taking the context into account.** They did this by completing an extensive review of the evidence on behaviors to address childhood weight management. **Second, Curtis et al selected the target behavior for the app to address.** They did this by conducting empirical research with the target population and consulted a paediatrician, dietician, two public health experts, and a family weight management service specialist about activities currently done and where the app could add value. **Third, Curtis et al. attempted to understand both the target behavior and user preferences** by conducting a qualitative study to address BCW and UCD methodologies. The study helped to understand barriers and facilitators to parents' capability, opportunity, and motivation in enacting the target behavior, as well as to explore parents' preferences for app features. The qualitative research involved conducting 6 focus groups, one with family weight management case workers and five with parents.

Define

the user and behavior change requirements

Summary of approaches and methods from 75 studies [1-75]

Methods
to "define"

- **Researcher synthesis** (i.e., research team makes sense of results)
- **Expert consensus** (i.e., clinical, technology and behavioral experts)
- **User prioritization** (i.e., patients and/or the public)

Key concepts to "Define"

DT

"User Requirements"

Define user centred requirements by analyzing and sorting user experiences and preferences into key themes and sub-themes.

Organization of Requirements

- Organize requirements by **shared** user beliefs & needs
- Create **user stories, journeys and scenarios**
- Translate user requirements into "**knowledge seeking questions**" for future ideation

BD

"Behavioral Requirements"

Make a “behavioral diagnosis” by analyzing empathy results, referring to constructs of a behavioral theory or model (e.g., COM-B).

Organization of Requirements

- Organize requirements by **behavioral determinants** that need to be addressed (e.g., motivation)
- **Select a behavioral model** to guide future design
- Translate requirements into a **logic model** that clarifies relevant behavioral outcomes, objectives and determinants

mHealth Specific Considerations

- Organize requirements by their **potential location in the mHealth application**
- Clarify whether requirements will be **structure or feature based** (structures are the technical elements to deliver the features)

Refine
requirements list

- Identify requirements within the **project scope and objectives**
- Use **feasibility criteria** to refine and select certain requirements
- **Consult experts and patient/ public users** to prioritize the requirements
- **Rank requirements** according to their (a) likelihood elicit behavior change, (b) alignment with current practices and guidelines, (c) adaptability to a digital interface, (d) acceptability to users, and (e) compatibility with data collection needs.

Define

the user and behavior change requirements

Examples from included studies [65,39,34,58]

Example 1:
Iterative four-phase development of a theory-based digital behavior change intervention to reduce occupational sedentary behavior by Stephenson et al., 2020 [65]

Stage 2 of Stephenson et al's design process involved **consensus workshops to amalgamate and discuss the findings of phase 1** (which included a systematic review, user focus groups and review of behavior change strategies), **gain expert opinion, and draw upon evidence from current literature. Mind-mapping** helped members define the requirements of the DBCI. **The APEASE criteria** (Acceptability, Practicability, Effectiveness and cost-effectiveness, Affordability, Safety/side-effects, Equity) helped members make sessions about which technology strategy would be most appropriate. The final selection of required intervention functions was informed by referring to (a) **the BCW framework to make a 'behavioral diagnosis'** (b) **existing apps and app features,** (c) **expert consensus on 'best bets'** based on previous findings from phase 1, and (d) **expert advice on feasibility in terms of computer programming.**

Example 2:
Co-Design of a Consultation Audio-Recording Mobile App for People With Cancer: The SecondEars App by Lipson-Smith et al., 2019 [39]

Lipson-Smith et al. relied on **iterative co-design methods the define and refine system requirements**. Co-design workshop attendees **continuously added to and refined a list of functions the app should be able to do.** Attendees helped craft key **'knowledge-seeking questions'** that would be used during ideation. **Journey Mapping** was also used to map out the pattern of use for the app in the context of the patients' journey. Workshop attendees then used **MoSCoW Methods to prioritize the apps 'must haves', 'should haves', 'could haves' and 'wont haves'** to reach a common understanding **on the scope of the project and the importance of previously brainstormed requirements.**

Example 3:
A Culturally Relevant Smartphone-Delivered Physical Activity Intervention for African American Women: Development and Initial Usability Tests of Smart Walk by Joseph et al., 2020 [34]

To analyze user focus group data (from Phase 1), Joseph et al. **created a codebook of primary deductive codes based on the five Social Cognitive Theory (SCT) constructs** (Behavioral Capacity, Outcome Expectations, Self-Efficacy, Social Support, and Self-Regulation). Secondary codes consisted of key concepts examined under each SCT construct. Joseph et al reviewed the coded data for repetitive themes within and across the specific SCT constructs. A local community member of the research team also reviewed the results. **Joseph et al presented findings according to the salient constructs of SCT, and outlined implications for how the findings could be translated and integrated into the design of Smart Walk.** The results also served to **clarify exactly how cultural norms and preferences of women** could be incorporated into the intervention design and delivery.

Example 4:
Development of a Smartphone Program to Support Adherence to Oral Chemotherapy in People with Cancer by Ross et al., 2019 [58]

Stage 2 of Ross et al's design process involved **clearly defining the technical features and structure of the smartphone program.** The selection of the **smartphone program's technical features and structure** was informed by findings from the **formative study** and a literature review (conducted by the research team) . The research team made sure to clearly define the **technical characteristics of the intervention** (eg, the platform used to deliver the strategies, personalization of the strategies) and **the frequency and conditions of intervention delivery** (eg, frequency of medication reminders) at this stage.

Ideate

user-centred features and behavior change content

Summary of approaches and methods from 75 studies [1-75]

Methods
to "Ideate"

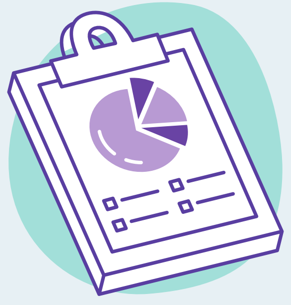

- **Researcher ideation** (i.e., researchers propose content and features)
- **Stakeholder workshops** (i.e., multidisciplinary team brainstorming)
- **Technology partner guidance** (i.e., app developers specify operationalization)
- **Patient/public engagement** (i.e., patient co-design and creative ideation)

Key approaches when "Ideating"

BD

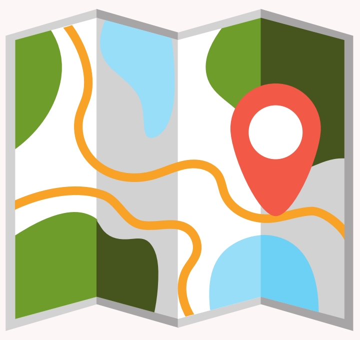

“Behavioral Mapping”

A structured, evidence-based approach to identify behavior change content

1. Select BCTs according to the behavioral diagnosis (e.g., utilize Michie's BCT Taxonomy)
2. Identify any additional behavioral “nudges” that look promising from other sources.
3. Translate BCTs and nudges into potential mHealth content and features
4. Reflect on how other applications have translated BCTs into mHealth features
5. Refine BCTs and nudges using feasibility criteria such as APEASE

Research-Led Ideation

- Researchers can create **mixed tables** to consider how behavior change insights and user preferences can both be translated into design themes and functionalities.
- Researchers can refer to a **"guiding principles"** document to reflect on how user requirements can be made compatible with the target behavior, theory, and a digital format.
- **Researchers can use their expertise** to reflect on how behavior change insights may be integrated with existing programs, practices and norms, as well as with best evidence from mHealth design and clinical innovation.

Technology-Partner Guidance

BD+DT

- **Technology partners can help iteratively ideate** and specify how behavioral and user requirements can be feasibility operationalized within an mHealth app (including design, functions, features and flows).
- **Tools such as the BIT model, PSD framework and/or Nielsen’s Heuristics** can be used to guide the translation of behavioral and user requirements into technical elements for the program.

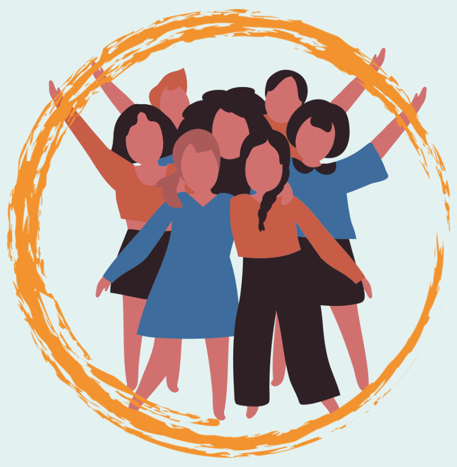

Multidisciplinary Stakeholder Workshops

- **Group discussion** about most important behavior change insights and strategies (including application content dose, delivery, and organization) suitable for the target population, a mobile app and behavior change objectives.
- **Brainstorm personalization and tailoring strategies** to deliver specific behavior change, user-centred and clinically relevant content.

Patient and Public Engagement

- **Co-designing** with patients/ the public can help ensure **behavior change insights** are user friendly.
- Getting patients/ the public to **rank, prioritize and conceptualize** how behavioral insights should be operationalized to meet their own needs.
- **Utilize visual and creative methods** to help users reflect on app ideas, content, and design.
- ·**Utilize “user personas”** to guide ideation, constantly checking if behavior change insights are accounted for (and to discuss their potential integration if not addressed)
- **Utilize “solution inspiration” and “white-boarding”** to structure how the app could be built.

Ideate

user-centred features and behavior change content

Examples from included studies [47,27,49]

Example 1:
Iterative development of Vegethon: a theory-based mobile app intervention to increase vegetable consumption by Mummah et al., 2016 [47]

After potential behavioral strategies were identified (from a literature review of behavior change strategies), **a multidisciplinary team engaged in a series of group brainstorming sessions,** also known as ideation, to conceive of **creative ways in which these techniques might be implemented.** Brainstorming centered around potential mobile app features and was inspired by a collection of **highly rated apps on the App Store**. A multidisciplinary team enabled the ideation of a wide range of diverse ideas **taking behavioral theory, user insights, and product experience into account.**

Example 2:
The development of Drink Less: an alcohol reduction smartphone app for excessive drinkers by Garnett et al., 2019 [27]

Garnett et al. translated specified intervention requirements into app modules. **First, BCTs targeting salient behavior constructs** were selected by the research team using a mapping approach guided by the BCT Taxonomy Version 1. **Second, the research team used PowerPoint slides** to describe how each intervention component could be translated into an app module (in terms of the text, graphics, and functionality) to the app developers. **The intervention components were translated into potential app modules in close collaboration with expert and experienced app developers.** Regular discussions between members of the research team and the developers were conducted to ensure that the description provided of the intervention components was translated in ways that were **feasible for users (judged by the app developers)** and **met the initial specification (judged by the research team).**

Example 3:
Development of an Intervention to Increase Sexual Health Service Uptake by Young People by Newby et al., 2019 [49]

First, behavior change techniques (BCTs) suitable for targeting the selected determinants were identified using the Behavior Change Wheel and BCT Taxonomy. The most appropriate BCTs for each determinant were chosen, excluding those that were either unfeasible given the digital mode of delivery or irrelevant to the target behavior. **Second, based on the system requirements and list of BCTs, ideas for the look, feel, and functionality of the app were developed by the steering group** (health psychologists, a public health consultant, a project manager, two young people, and a design lead) **and the Young People's Partnership Board (YPPB)** (group of end users). Over a period of 3 months, the steering group, the YPPB, and the design agency worked together to develop practical strategies (what users of the intervention would see and do) to deliver BCTs. This was a creative and iterative process. **All ideas were judged according to their feasibility and ability to effectively deliver the BCT.** The ability of the proposed ideas to engage the priority population was discussed among the YPPB. The steering group and YPPB made the final decision about which practical strategies should be used based on the above criteria.

Prototype

a solution that is user-centred and supports health behavior change

Summary of approaches and methods from 75 studies [1-75]

Methods to "Prototype"

- **Select a Prototyping "Approach"** (e.g., ,iterative rapid prototyping)
- **Utilize Prototyping "Tools"** (e.g., application flow charts and wire framing)
- **Consider a Protoptying "Aid"** (e.g., Persuasive System Design framework)

Key Prototyping Approaches

DT

BD

Design Thinking Approach

Iterative prototyping, feedback and refinement (e.g., using sprint, scrum and agile methods)

↔

Behavioral Design Considerations

Co-Development of BCTs: Users and key stakeholders can help clarify what (content), when (frequency), how many (dose), who (target user) and which (medium type) BCTs should be delivered. Co-development can also help create more appropriate questions to personalize application content (to deliver unique behavioral, clinical and user-centred content).

Develop an Intervention Plan: Developing an "Intervention Plan" can help clarify the reasons behind the ideated content (i.e., behavioral and user-centred linkages) which may help ensure BCTs and user preferences are not lost in translation during technological development.

Key Prototyping Tools

Design Thinking Tools

Wire framing, paper prototyping, rapid mock-ups, system architecture diagrams, app flow charts, user stories, and use case scenarios.

↔

Behavioral Design Considerations

BCT Flow Charts: Map out how the BCTs will be interacted within the proposed prototype.

User Stories organized by Target Behavior Changes: Organize user stories by the target behavior changes to be achieved to ensure the solution meets relevant behavioral requirements.

Key Prototyping Aids

Design Thinking Aids

Usability Heuristics, Eight Golden Rules, Human Interface Guidelines

↔

Behavioral Design Considerations

Use the PSD Framework, BIT Model or Computer Tailoring Method: Using a behaviorally-informed technology development framework or model can help guide the translation of behavioral insights into operationalized features and functions within a prototype.

Prototype

a solution that is user-centred and supports behavior change

Examples from included studies [69,16,64,68]

Example 1:
Using codesign to develop a culturally tailored, behavior change mHealth intervention for indigenous and other priority communities: A case study in New Zealand by Verbiest et al., 2018 [69]

Prototyping was an iterative process for Verbiest et al., with regular feedback and consensus meetings. The app developer took the high level of input from the community focus groups and worked with the multidisciplinary research team to create several wireframe prototypes of the mHealth tool. **Incorporating BCTs into mHealth intervention was an iterative process founded on community based co-design.** Through the co-design and early prototyping, it became evident that despite community partners having similar ideas with regard to BCTs, there were clear differences with regard to the content of them and how they would be delivered. Communities provided feedback on the wireframes and broad consensus was reached regarding content, features, and functionalities (i.e., not everything could be adopted due to reasons such as time and technology constraints).

Example 2:
Targeting Parents for Childhood Weight Management: Development of a Theory-Driven and User-Centered Healthy Eating App by Curtis et al., 2015 [16]

After deciding what BCTs to include, Curtis et al aimed to **embed these BCTs into an actual app** informed by user preferences. **Consulting with the app company facilitated** the process of how BCTs identified in previous steps could be meaningfully combined with findings on user preferences **to create app features.** Consultations with a software engineer led to the development of a **"Flow Chart" to map specific BCTs to app features**, showing the sequence of how BCTs would be delivered to parents. The diagram was further refined through parental feedback. **With respect to enhancing user experience and engagement in the application**, parents' preferences for app features were combined with suggestions from the app company. For example, progress bars, achievement badges, and points were identified as a way of providing feedback for parents on their children's eating behavior.

Example 3:
The Design and Development of a Personalized Leisure Time Physical Activity Application Based on Behavior Change Theories, End-User Perceptions, and Principles From Empirical Data Mining by Sporrel et al., 2021 [64]

Sporrel et al. outlined that while some frameworks provide a step-wise instruction to develop an mHealth intervention (e.g., IDEAS framework) others provide guidance for specific steps such as the translation of behavioral theories to persuasive strategies delivered within an app **(e.g., BIT model)**. Sporrel et al. used the **BIT model to guide the practical implementation and design** of the persuasive strategies in the app. Specifically, the BIT model helped to describe **what intervention modules the user receives** (e.g., notifications), **how it relates to the persuasive strategies** (e.g., reminders), **when they have access to the module** (e.g., under which condition), and **how the modules look like** (e.g., complexity and aesthetics). The team outlined these conditions by **creating "User Stories"** with stakeholders, behavioral scientists, computer scientists, and the app developer**.** The "user stories" served to translate the requirements into the app modules, as **they explain the technical implementation and design characteristics of the selected persuasive strategy** (e.g., "As a user, I want to restrict the amount of messages I receive during the week, so I do not get disturbed too frequently and get annoyed by the intervention.")

Example 4:
Kick.it: The development of an evidence-based smoking cessation smartphone app by van Agteren et al., 2018 [68]

van Agteren et al argued that although the design process steps 1–3 resulted in the selection of evidence-based BCTs and practical applications to accomplish behavioral change, they **did not provide sufficient guidance on the design of mHealth interventions.** Using the Persuasive System Design (PSD) framework helped can Agteren guide the overall functional development of Kick.it. The PSD framework provides intervention designers with **28 design principles** falling into four categories that aim to **ensure that the final system is actually capable of supporting sustainable health behavior change**.

Test

solution against user needs and for its behavior change potential

Summary of approaches and methods from 75 studies [1-75]

DT

BD

Heuristic Evaluation

Design Thinking Testing

Heuristic Evaluation: Assess the intervention for the presence of Nielsen's 10 principles

Behavioral Design Considerations

BCT Evaluation: Assess the intervention for the presence of BCTs and other nudges (using a pre-defined taxonomy)

User Testing

Design Thinking Testing

Usability Testing: Test the solution for its usability, functionality, acceptability, satisfaction, usefulness, and value. Track user comments, behavior, error rates and task completion time during testing.
Testing can utilize Cognitive Walkthroughs, Think Aloud Methods, Participant Debrief, User Interview and Focus Groups, User Surveys (often using SUS Scale, MARS Scale, UTAUT Scale or the Five Dimensions of Usability).

Behavioral Design Considerations

BCT Usability Testing: Tag components of the intervention that utilize BCTs, and follow how users engage with them, understand their acceptability, and assess their perceived usefulness and value.
Behaviorally Informed User Interview Guides: Use behavioral theories/ models to develop interview guides to assess intervention's favourability to address specific behavioral determinants.

Expert Evaluation

Design Thinking Testing

Expert Evaluation: Expert perspectives can be elicited to assess appropriateness for target population, cultural sensitivity, message appeal, message readability, potential effectiveness, and technical feasibility (i.e., health providers, communication specialists, behavior change specialists, patient experts, etc.). Expert evaluation can be done using expert advisory panel meetings, expert consensus methods, health literacy evaluations, and content fidelity assessments.

Behavioral Design Considerations

BCT Fidelity Test: Have behavior change experts assess the extent to which the intervention content has fidelity to the intended BCTs.
App Behavior Change Scale (ABACUS) Evaluation: Assess the quality of the behavior change application and its potential to facilitate behavior change using ABACUS.
"Engagement" Definition: Assess how experts conceptualize "engagement" in the context of the specific mHealth DBCI.

Pilot Testing

Design Thinking Testing

Pilot with users: Roll out the application for a short period of time to select members of the target population. Track usage behaviors, identify bugs/defects in implementation, conduct debrief interviews, and administer surveys to measure usefulness, attractiveness, and ease of use (and other measures/ scale).

Behavioral Design Considerations

Change in knowledge: understanding of health content
Behavioral intentions: app use and health behavior
State of change: using Transtheoretical Model (TTM)
User experience with BCTs: acceptance of BCTs
Perceived potential of BCTs: beleifs on efficacy of BCTs
User engagement with BCTs: Tracking use of BCTs
Random assignment to BCTs: allocation to BCTs

Test

the solution against user needs and for its behavior change potential

Examples from included studies [45,42,27,44,26]

Example 1:
Details of development of the resource for adults with asthma in the RAISIN (randomized trial of an asthma internet self-management intervention) study by Morrison et al., 2015 [45]

The final version of the intervention was **formally mapped to Michie and colleagues latest BCT taxonomy** in order to describe which BCTs were present. Every page was reviewed (and where a BCT was explicitly meant to be assigned). Morrison et al did this evaluation to provide a reliable record of the content of the behavior change intervention, and to confirm that they included the BCTs as planned.

Example 2:
Development and Acceptability of a Tablet-Based App to Support Men to Link to HIV Care: Mixed Methods Approach by Mathenjwa et al., 2020 [42]

Mathenjwa et al completed **in-depth user interviews** with participants who received the intervention. The interview questions explored why participants had not been linked to care after the HIV diagnosis and their views of the intervention and the impact it had on them**. Interviews were thematically analyzed with themes guided by Self-Determination Theory (SDT), to understand the intervention's potential impact on autonomy, competence, and relatedness.**

Example 3:
The development of Drink Less: an alcohol reduction smartphone app for excessive drinkers by Garnett et al., 2019 [27]

Garnett et al. conducted a **usability testing study** to explore user views toward the app in order to **determine whether the BCTs were acceptable and feasible to users and how they might be improved**. The usability study consisted of two parts: **a think aloud study** to understand users' first impressions and **semistructured interviews** to investigate user's impressions of using the intervention in naturalistic settings.

Example 4:
A Personalized Physical Activity Coaching App for Breast Cancer Survivors: Design Process and Early Expert Evaluationby Moneiro-Guerra et al., 2020 [44]

An expert evaluation was performed with the mobile app rating scale (MARS) and the app behavior change scale (ABACUS) to assess the quality of the app and the potential for behavior change, respectively. The MARS examined app elements such as engagement, functionality, utility, aesthetics, and information. An overall functionality score out of 5 was derived using this scale. The ABACUS scale comprises 21 items and was used to examine the potential behavior change of the app in relation to goal setting, action planning, barrier identification, self-monitoring, and feedback.

Example 5:
Design of a Mobile App for Nutrition Education (TreC-LifeStyle) and Formative Evaluation With Families of Overweight Children by Gabrielli et al., 2017 [26]

Gabrielli at al conducted a pilot test of their intervention, and assessed: (1) **parents' knowledge** of the Mediterranean Diet guidelines; (2) **state of change** for healthy nutrition-measured by an adapted version of the URICA-short-form scale, based on the 4 readiness to change states of the Transtheoretical Model of Change, and (3) **intention to use** technology for nutrition education. **After 3 weeks of the app usage,** any changes on the above factors were recorded and parents' perceived usability of the app was assessed (System Usability Scale Questionnaire). **After 6 weeks of app usage**, any changes in the factors above were recorded again as well as a more in depth review of the participants' experience with the mHealth intervention in a semi-structured interview.

References

[1] Adu, M. D., Malabu, U. H., Malau-Aduli, A. E. O., & Malau-Aduli, B. S. (2020). The development of My Care Hub mobile-phone app to support self-management in Australians with type 1 or type 2 diabetes. Scientific Reports, 10(1), 1–10.
[2] Antypas, K., & Wangberg, S. C. (2014). Combining users' needs with health behavior models in designing an internet-and mobile-based intervention for physical activity in cardiac rehabilitation. JMIR Research Protocols, 3(1), e4.
[3] Asbjørnsen, R. A., Wentzel, J., Smedsrød, M. L., Hjelmesæth, J., Clark, M. M., Nes, L. S., & Van Gemert-Pijnen, J. E. W. C. (2020). Identifying persuasive design principles and behavior change techniques supporting end user values and needs in ehealth interventions for long-term weight loss maintenance: qualitative study. Journal of Medical Internet Research, 22(11), e22598.
[4] Athilingam, P., Clochesy, J. M., & Labrador, M. A. (2018). Intervention mapping approach in the design of an interactive mobile health application to improve self-care in heart failure. CIN: Computers, Informatics, Nursing, 36(2), 90–97.
[5] Bartlett, Y. K., Farmer, A., Rea, R., & French, D. P. (2020). Use of brief messages based on behavior change techniques to encourage medication adherence in people with type 2 diabetes: developmental studies. Journal of Medical Internet Research, 22(5), e15989.
[6] Beentjes, T. A. A., van Gaal, B. G. I., Goossens, P. J. J., & Schoonhoven, L. (2015). Development of an e-supported illness management and recovery programme for consumers with severe mental illness using intervention mapping, and design of an early cluster randomized controlled trial. BMC Health Services Research, 16(1), 1–9.
[7] Bogale, B., Mørkrid, K., O'Donnell, B., Ghanem, B., Ward, I. A., Khader, K. A., Isbeih, M., Frost, M., Baniode, M., & Hijaz, T. (2020). Development of a targeted client communication intervention to women using an electronic maternal and child health registry: a qualitative study. BMC Medical Informatics and Decision Making, 20(1), 1–12.
[8] Brendryen, H., Johansen, A. B., Nesvåg, S., Kok, G., & Duckert, F. (2013). Constructing a theory-and evidence-based treatment rationale for complex eHealth interventions: Development of an online alcohol intervention using an intervention mapping approach. JMIR Research Protocols, 2(1), e6.
[9] Brown, J. M., Savaglio, R., Watson, G., Kaplansky, A., LeSage, A., Hughes, J., Kapralos, B., & Arcand, J. (2020). Optimizing child nutrition education with the Foodbot Factory mobile health app: formative evaluation and analysis. JMIR Formative Research, 4(4), e15534.
[10] Buman, M. P., Epstein, D. R., Gutierrez, M., Herb, C., Hollingshead, K., Huberty, J. L., Hekler, E. B., Vega-López, S., Ohri-Vachaspati, P., & Hekler, A. C. (2016). BeWell24: development and process evaluation of a smartphone "app" to improve sleep, sedentary, and active behaviors in US Veterans with increased metabolic risk. Translational Behavioral Medicine, 6(3), 438–448.
[11] Carter, L., Rogith, D., Franklin, A., & Myneni, S. (2019). NewCope: A Theory-Linked Mobile Application for Stress Education and Management. Studies in Health Technology and Informatics, 264, 1150.
[12] Chang, W.-J., Lo, S.-Y., Kuo, C.-L., Wang, Y.-L., & Hsiao, H.-C. (2019). Development of an intervention tool for precision oral self-care: Personalized and evidence-based practice for patients with periodontal disease. PloS One, 14(11), e0225453.
[13] Chen, J., Ho, E., Jiang, Y., Whittaker, R., Yang, T., & Bullen, C. (2020). Mobile Social Network–Based Smoking Cessation Intervention for Chinese Male Smokers: Pilot Randomized Controlled Trial. JMIR MHealth and UHealth, 8(10), e17522.
[14] Chen, Y., Wu, F., Wu, Y., Li, J., Yue, P., Deng, Y., Lamb, K. V, Fong, S., Liu, Y., & Zhang, Y. (2019). Development of interventions for an intelligent and individualized mobile health care system to promote healthy diet and physical activity: using an intervention mapping framework. BMC Public Health, 19(1), 1–16.
[15] Coumans, J. M. J., Bolman, C. A. W., Friederichs, S. A. H., Oenema, A., & Lechner, L. (2020). Development and testing of a personalized web-based diet and physical activity intervention based on motivational interviewing and the self-determination theory: protocol for the mylifestylecoach randomized controlled trial. JMIR Research Protocols, 9(2), e14491.

[continued below]

References [continued]

[16] Curtis, K. E., Lahiri, S., & Brown, K. E. (2015). Targeting parents for childhood weight management: development of a theory-driven and user-centered healthy eating app. JMIR MHealth and UHealth, 3(2), e3857.
[17] Dalum, P., Brandt, C. L., Skov-Ettrup, L., Tolstrup, J., & Kok, G. (2016). The systematic development of an internet-based smoking cessation intervention for adults. Health Promotion Practice, 17(4), 490–500.
[18] den Bakker, C. M., Schaafsma, F. G., van der Meij, E., Meijerink, W. J. H. J., van den Heuvel, B., Baan, A. H., Davids, P. H. P., Scholten, P. C., van der Meij, S., & van Baal, W. M. (2019). Electronic health program to empower patients in returning to normal activities after general surgical and gynecological procedures: Intervention mapping as a useful method for further development. Journal of Medical Internet Research, 21(2), e9938.
[19] DeSmet, A., De Bourdeaudhuij, I., Chastin, S., Crombez, G., Maddison, R., & Cardon, G. (2019). Adults' preferences for behavior change techniques and engagement features in a mobile app to promote 24-hour movement behaviors: Cross-sectional survey study. JMIR MHealth and UHealth, 7(12), e15707.
[20] Direito, A., Walsh, D., Hinbarji, M., Albatal, R., Tooley, M., Whittaker, R., & Maddison, R. (2018). Using the intervention mapping and behavioral intervention technology frameworks: development of an mHealth intervention for physical activity and sedentary behavior change. Health Education & Behavior, 45(3), 331–348.
[21] Duff, O., Walsh, D., Malone, S., McDermott, L., Furlong, B., O'Connor, N., Moran, K., & Woods, C. (2018). MedFit app, a behavior-changing, theoretically informed mobile app for patient self-management of cardiovascular disease: user-centered development. JMIR Formative Research, 2(1), e9550.
[22] Edwards, E. A., Caton, H., Lumsden, J., Rivas, C., Steed, L., Pirunsarn, Y., Jumbe, S., Newby, C., Shenvi, A., & Mazumdar, S. (2018). Creating a theoretically grounded, gamified health app: Lessons from developing the Cigbreak smoking cessation mobile phone game. JMIR Serious Games, 6(4), e10252.
[23] Engelen, M. M., van Dulmen, S., Puijk-Hekman, S., Vermeulen, H., Nijhuis-van der Sanden, M. W., Bredie, S. J., & van Gaal, B. G. (2020). Evaluation of a Web-Based Self-Management Program for Patients With Cardiovascular Disease: Explorative Randomized Controlled Trial. Journal of Medical Internet Research, 22(7), e17422. https://doi.org/https://dx.doi.org/10.2196/17422
[24] Félix, I. B., Guerreiro, M. P., Cavaco, A., Cláudio, A. P., Mendes, A., Balsa, J., Carmo, M. B., Pimenta, N., & Henriques, A. (2019). Development of a complex intervention to improve adherence to antidiabetic medication in older people using an anthropomorphic virtual assistant software. Frontiers in Pharmacology, 10, 680.
[25] Fuemmeler, B. F., Holzwarth, E., Sheng, Y., Do, E. K., Miller, C. A., Blatt, J., Rosoff, P. M., & Østbye, T. (2020). Mila blooms: a mobile phone application and behavioral intervention for promoting physical activity and a healthy diet among adolescent survivors of childhood cancer. Games for Health Journal, 9(4), 279–289.
[26] Gabrielli, S., Dianti, M., Maimone, R., Betta, M., Filippi, L., Ghezzi, M., & Forti, S. (2017). Design of a mobile app for nutrition education (TreC-LifeStyle) and formative evaluation with families of overweight children. JMIR MHealth and UHealth, 5(4), e7080.
[27] Garnett, C., Crane, D., West, R., Brown, J., & Michie, S. (2019). The development of Drink Less: an alcohol reduction smartphone app for excessive drinkers. Translational Behavioral Medicine, 9(2), 296–307.
[28] Giunti, G., Mylonopoulou, V., & Romero, O. R. (2018). More stamina, a gamified mhealth solution for persons with multiple sclerosis: research through design. JMIR MHealth and UHealth, 6(3), e9437.
[29] Hales, S., Turner-McGrievy, G., Fahim, A., Freix, A., Wilcox, S., Davis, R. E., Huhns, M., & Valafar, H. (2016). A mixed-methods approach to the development, refinement, and pilot testing of social networks for improving healthy behaviors. JMIR Human Factors, 3(1), e4512.
[30] Hawley-Hague, H., Tacconi, C., Mellone, S., Martinez, E., Ford, C., Chiari, L., Helbostad, J., & Todd, C. (2020). Smartphone apps to support falls rehabilitation exercise: app development and usability and acceptability study. JMIR MHealth and UHealth, 8(9), e15460.

[continued below]

References [continued]

[31] Hebden, L., Cook, A., Van Der Ploeg, H. P., & Allman-Farinelli, M. (2012). Development of smartphone applications for nutrition and physical activity behavior change. JMIR Research Protocols, 1(2), e2205.
[32] Hendrie, G. A., James-Martin, G., Williams, G., Brindal, E., Whyte, B., & Crook, A. (2019). The development of VegEze: smartphone app to increase vegetable consumption in Australian adults. JMIR Formative Research, 3(1), e10731.
[33] Hooglugt, F., & Ludden, G. D. S. (2020). A mobile app adopting an identity focus to promote physical activity (MoveDaily): iterative design study. JMIR MHealth and UHealth, 8(6), e16720.
[34] Joseph, R. P., Keller, C., Vega-López, S., Adams, M. A., English, R., Hollingshead, K., Hooker, S. P., Todd, M., Gaesser, G. A., & Ainsworth, B. E. (2020). A culturally relevant smartphone-delivered physical activity intervention for African American women: Development and initial usability tests of smart walk. JMIR MHealth and UHealth, 8(3), e15346.
[35] Kazemi, D. M., Borsari, B., Levine, M. J., Lamberson, K. A., & Dooley, B. (2018). REMIT: Development of a mHealth theory-based intervention to decrease heavy episodic drinking among college students. Addiction Research & Theory, 26(5), 377–385. https://doi.org/https://dx.doi.org/10.1080/16066359.2017.1420783
[36] Kim, B., Lee, D., Min, A., Paik, S., Frey, G., Bellini, S., Han, K., & Shih, P. C. (2020). PuzzleWalk: A theory-driven iterative design inquiry of a mobile game for promoting physical activity in adults with autism spectrum disorder. Plos One, 15(9), e0237966.
[37] Korpershoek, Y. J. G., Hermsen, S., Schoonhoven, L., Schuurmans, M. J., & Trappenburg, J. C. A. (2020). User-centered design of a mobile health intervention to enhance exacerbation-related self-management in patients with chronic obstructive pulmonary disease (copilot): mixed methods study. Journal of Medical Internet Research, 22(6), e15449.
[38] Lee, M. K., Park, H.-A., Yun, Y. H., & Chang, Y. J. (2013). Development and formative evaluation of a web-based self-management exercise and diet intervention program with tailored motivation and action planning for cancer survivors. JMIR Research Protocols, 2(1), e2331.
[39] Lipson-Smith, R., White, F., White, A., Serong, L., Cooper, G., Price-Bell, G., & Hyatt, A. (2019). Co-design of a consultation audio-recording mobile app for people with cancer: the SecondEars app. JMIR Formative Research, 3(1), e11111.
[40] Maidment, D. W., Coulson, N. S., Wharrad, H., Taylor, M., & Ferguson, M. A. (2020). The development of an mHealth educational intervention for first-time hearing aid users: Combining theoretical and ecologically valid approaches. International Journal of Audiology, 59(7), 492–500.
[41] Mann, D. M., Quintiliani, L. M., Reddy, S., Kitos, N. R., & Weng, M. (2014). Dietary approaches to stop hypertension: lessons learned from a case study on the development of an mHealth behavior change system. JMIR MHealth and UHealth, 2(4), e41.
[42] Mathenjwa, T., Adeagbo, O., Zuma, T., Dikgale, K., Zeitlin, A., Matthews, P., Seeley, J., Wyke, S., Tanser, F., & Shahmanesh, M. (2020). Development and Acceptability of a Tablet-Based App to Support Men to Link to HIV Care: Mixed Methods Approach. JMIR MHealth and UHealth, 8(11), e17549.
[43] McCarthy, O. L., Wazwaz, O., Calderon, V. O., Jado, I., Saibov, S., Stavridis, A., Gallardo, J. L., Tokhirov, R., Adada, S., & Huaynoca, S. (2018). Development of an intervention delivered by mobile phone aimed at decreasing unintended pregnancy among young people in three lower middle income countries. BMC Public Health, 18(1), 1–15.
[44] Monteiro-Guerra, F., Signorelli, G. R., Tadas, S., Zubiete, E. D., Romero, O. R., Fernandez-Luque, L., & Caulfield, B. (2020). A personalized physical activity coaching app for breast cancer survivors: design process and early prototype testing. JMIR MHealth and UHealth, 8(7), e17552.
[45] Morrison, D., Mair, F. S., Chaudhuri, R., McGee-Lennon, M., Thomas, M., Thomson, N. C., Yardley, L., & Wyke, S. (2015). Details of development of the resource for adults with asthma in the RAISIN (randomized trial of an asthma internet self-management intervention) study. BMC Medical Informatics and Decision Making, 15(1), 1–16.

[continued below]

References [continued]

[46] Mueller, J., Davies, A., Jay, C., Harper, S., Blackhall, F., Summers, Y., Harle, A., & Todd, C. (2019). Developing and testing a web-based intervention to encourage early help-seeking in people with symptoms associated with lung cancer. British Journal of Health Psychology, 24(1), 31–65.
[47] Mummah, S. A., King, A. C., Gardner, C. D., & Sutton, S. (2016). Iterative development of Vegethon: a theory-based mobile app intervention to increase vegetable consumption. International Journal of Behavioral Nutrition and Physical Activity, 13(1), 1–12.
[48] Nelson, L. A., Mayberry, L. S., Wallston, K., Kripalani, S., Bergner, E. M., & Osborn, C. Y. (2016). Development and usability of REACH: a tailored theory-based text messaging intervention for disadvantaged adults with type 2 diabetes. JMIR Human Factors, 3(2), e6029.
[49] Newby, K. V, Brown, K. E., Bayley, J., Kehal, I., Caley, M., Danahay, A., Hunt, J., & Critchley, G. (2017). Development of an intervention to increase sexual health service uptake by young people. Health Promotion Practice, 18(3), 391–399.
[50] Partridge, S. R., Raeside, R., Latham, Z., Singleton, A. C., Hyun, K., Grunseit, A., Steinbeck, K., & Redfern, J. (2019). 'Not to Be Harsh but Try Less to Relate to "the Teens" and You'll Relate to Them More': Co-Designing Obesity Prevention Text Messages with Adolescents. International Journal of Environmental Research and Public Health, 16(24), 4887.
[51] Peleg, M., Michalowski, W., Wilk, S., Parimbelli, E., Bonaccio, S., O'Sullivan, D., Michalowski, M., Quaglini, S., & Carrier, M. (2018). Ideating mobile health behavioral support for compliance to therapy for patients with chronic disease: a case study of atrial fibrillation management. Journal of Medical Systems, 42(11), 1–15.
[52] Potzel, A. L., Gar, C., Seissler, J., & Lechner, A. (2021). A Smartphone App (TRIANGLE) to Change Cardiometabolic Risk Behaviors in Women Following Gestational Diabetes Mellitus: Intervention Mapping Approach. JMIR MHealth and UHealth, 9(5), e26163.
[53] Quintiliani, L. M., Foster, M., & Oshry, L. J. (2019). Preferences of mHealth app features for weight management among breast cancer survivors from underserved populations. Psycho-Oncology, 28(10), 2101.
[54] Reidy, C., Foster, C., & Rogers, A. (2020). A facilitated web-based self-management tool for people with type 1 diabetes using an insulin pump: intervention development using the behavior change wheel and theoretical domains framework. Journal of Medical Internet Research, 22(5), e13980.
[55] Ribaut, J., Leppla, L., Teynor, A., Valenta, S., Dobbels, F., Zullig, L. L., & De Geest, S. (2020). Theory-driven development of a medication adherence intervention delivered by eHealth and transplant team in allogeneic stem cell transplantation: the SMILe implementation science project. BMC Health Services Research, 20(1), 1–22.
[56] Rodrigues, A. M., Sniehotta, F. F., Birch-Machin, M. A., Olivier, P., & Araújo-Soares, V. (2017). Systematic and iterative development of a smartphone app to promote sun-protection among holidaymakers: design of a prototype and results of usability and acceptability testing. JMIR Research Protocols, 6(6), e112.
[57] Rohde, A., Duensing, A., Dawczynski, C., Godemann, J., Lorkowski, S., & Brombach, C. (2019). An app to improve eating habits of adolescents and young adults (challenge to go): systematic development of a theory-based and target group–adapted mobile app intervention. JMIR MHealth and UHealth, 7(8), e11575.
[58] Ross, X. S., Gunn, K. M., Patterson, P., & Olver, I. (2019). Development of a smartphone program to support adherence to oral chemotherapy in people with cancer. Patient Preference and Adherence, 13, 2207.
[59] Sakakibara, B. M., Lear, S. A., Barr, S. I., Benavente, O., Goldsmith, C. H., Silverberg, N. D., Yao, J., & Eng, J. J. (2017). Development of a chronic disease management program for stroke survivors using intervention mapping: the stroke coach. Archives of Physical Medicine and Rehabilitation, 98(6), 1195–1202.
[60] Scheerman, J. F. M., Van Empelen, P., Van Loveren, C., & Van Meijel, B. (2018). A mobile app (WhiteTeeth) to promote good oral health behavior among Dutch adolescents with fixed orthodontic appliances: intervention mapping approach. JMIR MHealth and UHealth, 6(8), e163.

[continued below]

References [continued]

[61] Shoneye, C. L., Mullan, B., Begley, A., Pollard, C. M., Jancey, J., & Kerr, D. A. (2020). Design and Development of a Digital Weight Management Intervention (ToDAy): Qualitative Study. JMIR MHealth and UHealth, 8(9), e17919.
[62] Simons, D., De Bourdeaudhuij, I., Clarys, P., De Cocker, K., Vandelanotte, C., & Deforche, B. (2018). A smartphone app to promote an active lifestyle in lower-educated working young adults: development, usability, acceptability, and feasibility study. JMIR MHealth and UHealth, 6(2), e8287.
[63] Sittig, S., Wang, J., Iyengar, S., Myneni, S., & Franklin, A. (2020). Incorporating behavioral trigger messages into a mobile health app for chronic disease management: randomized clinical feasibility trial in diabetes. JMIR MHealth and UHealth, 8(3), e15927.
[64] Sporrel, K., De Boer, R. D. D., Wang, S., Nibbeling, N., Simons, M., Deutekom, M., Ettema, D., Castro, P. C., Dourado, V. Z., & Kröse, B. (2020). The Design and Development of a Personalized Leisure Time Physical Activity Application Based on Behavior Change Theories, End-User Perceptions, and Principles From Empirical Data Mining. Frontiers in Public Health, 8.
[65] Stephenson, A., Garcia-Constantino, M., McDonough, S. M., Murphy, M. H., Nugent, C. D., & Mair, J. L. (2020). Iterative four-phase development of a theory-based digital behaviour change intervention to reduce occupational sedentary behaviour. Digital Health, 6, 2055207620913410.
[66] Toefy, Y., Skinner, D., & Thomsen, S. (2016). “Please Don’t Send Us Spam!” A Participative, Theory-Based Methodology for Developing an mHealth Intervention. JMIR MHealth and UHealth, 4(3), e6041.
[67] Tonkin, E., Jeffs, L., Wycherley, T. P., Maher, C., Smith, R., Hart, J., Cubillo, B., & Brimblecombe, J. (2017). A smartphone app to reduce sugar-sweetened beverage consumption among young adults in Australian remote indigenous communities: design, formative evaluation and user-testing. JMIR MHealth and UHealth, 5(12), e8651.
[68] van Agteren, J. E. M., Lawn, S., Bonevski, B., & Smith, B. J. (2018). Kick. it: the development of an evidence-based smoking cessation smartphone app. Translational Behavioral Medicine, 8(2), 243–267.
[69] Verbiest, M. E. A., Corrigan, C., Dalhousie, S., Firestone, R., Funaki, T., Goodwin, D., Grey, J., Henry, A., Humphrey, G., & Jull, A. (2019). Using codesign to develop a culturally tailored, behavior change mHealth intervention for indigenous and other priority communities: A case study in New Zealand. Translational Behavioral Medicine, 9(4), 720–736.
[70] Walsh, D. M. J., Moran, K., Cornelissen, V., Buys, R., Claes, J., Zampognaro, P., Melillo, F., Maglaveras, N., Chouvarda, I., & Triantafyllidis, A. (2019). The development and codesign of the PATHway intervention: a theory-driven eHealth platform for the self-management of cardiovascular disease. Translational Behavioral Medicine, 9(1), 76–98.
[71] Warren, I., Meads, A., Whittaker, R., Dobson, R., & Ameratunga, S. (2018). Behavior change for youth drivers: design and development of a smartphone-based app (BackPocketDriver). JMIR Formative Research, 2(2), e9660.
[72] Waterlander, W., Whittaker, R., McRobbie, H., Dorey, E., Ball, K., Maddison, R., Smith, K. M., Crawford, D., Jiang, Y., & Gu, Y. (2014). Development of an evidence-based mHealth weight management program using a formative research process. JMIR MHealth and UHealth, 2(3), e2850.
[73] Wen, K.-Y., Miller, S. M., Kilby, L., Fleisher, L., Belton, T. D., Roy, G., & Hernandez, E. (2014). Preventing postpartum smoking relapse among inner city women: development of a theory-based and evidence-guided text messaging intervention. JMIR Research Protocols, 3(2), e3059.
[74] White, B. K., Martin, A., White, J. A., Burns, S. K., Maycock, B. R., Giglia, R. C., & Scott, J. A. (2016). Theory-based design and development of a socially connected, gamified mobile app for men about breastfeeding (Milk Man). JMIR MHealth and UHealth, 4(2), e5652.
[75] Zuidema, R. M., van Gaal, B. G. I., van Dulmen, S., Repping-Wuts, H., & Schoonhoven, L. (2015). An online tailored self-management program for patients with rheumatoid arthritis: a developmental study. JMIR Research Protocols, 4(4), e140.

[end]
