# Supplementary material for: Integrating Behavioral Science and Design Thinking to Develop Mobile Health Interventions: Systematic Scoping Review
Source: JMIR Mhealth Uhealth. 2022 Mar 16;10(3):e35799. doi: 10.2196/35799 (PMC8968622; doi:10.2196/35799)
Supplement: Multimedia Appendix 7 [file mhealth_v10i3e35799_app7.doc]

**Multimedia Appendix 7: Implementation Considerations, Design Challenges, and Future Directions**

| **Challenges with mHealth DBCI design** | |
| --- | --- |
| Time and resource consuming | The design process can be time and resource intensive, especially when design process best-practices are unclear. |
| Lack of meaningful involvement | It can be difficult to recruit representative end-users and involve them throughout the design process. |
| Conflicting evidence and needs | There is often a conflict between behavioral theory, user preferences, and stakeholder recommendations. Even if content is agreed on, there is often a gap between intervention developer conceptualization and software developer operationalization. |
| BCT translation into mHealth | It can often be unclear or complex to operationalize BCTs in the mHealth platform. Issues include; (1) too many BCTs may increase complexity, (2) attempting to integrate BCTs may crowd out creativity, (3) operationalized BCTs may lack fidelity to original concept, (4) BCTs may be prioritized, combined, and tailored inappropriately. |
| Lack of evaluation rigor | It can be difficult to evaluate the mHealth DBCI in a meaningful way; Issues include; (1) recruit appropriate participants, (2) lack of rigorous testing, (3) lack of ability to test for behavior change outcomes. |
| Clinical integration | It can be difficult to meaningfully integrate the mHealth DBCI within the local healthcare context. Issues include; (1) Practitioner gatekeeping, (2) Ethical and privacy issues, (3) Budgeting, (4) Managing expectations. |
| mHealth limitations | Delivering interventions on an mHealth platform come with their own challenges. These include (1) inability of mHealth to address environmental barriers, (2) Inequity considerations, (3) Sustainability of mHealth platform overtime. |
|  |  |
| **Implementation considerations** | |
| Create an implementation plan | Create an implementation plan that addresses issues regarding advertising, promotion, dissemination, onboarding, adoption, usage, and sustainability. Models of user engagement may be referred to. |
| Conduct implementation evaluations | Consider testing for potential implementation barriers and facilitators within the design process |
| Use feasibility criteria throughout design | Use feasibility criteria throughout the design process to ensure all ideas will be implementable in practice. |
| Ensure stakeholder buy-in and clinical integration | Involve future implementers (eg, patients, clinicians, etc.) early in the design process to ensure clinical and patient integration in the broader healthcare experience. |
|  |  |
| **Future directions for mHealth DBCI design** | |
| Design process description | Clear direction on the mHealth DBCI design process. Evaluating best practices in design could help clarify what works and why. |
| BCT representation in mHealth | Clear direction on how BCTs can be represented in mHealth in a meaningful and user-friendly way. |
| Tailoring and personalization | It is unlikely that a magic bullet solution exists to mHealth DBCI design. Allowing for user tailoring and personalization will be important. |
| Meaningful involvement of stakeholders in design | Clear direction on how to meaningfully involve end-users and stakeholders in mHealth DBCI design. This includes how to recruit and engagement these partners throughout. |
| Sustainability considerations | Although initial implementation and adoption of the mHealth DBCI may be supported, long term sustainability of the platform and its usage may be problematic. Understanding determinants of long term sustainability for mHealth DBCIs in practice and by users should be considered. |
